# *Undaria pinnatifida*: A case study to highlight challenges in marine invasion ecology and management

**DOI:** 10.1002/ece3.3430

**Published:** 2017-09-22

**Authors:** Graham Epstein, Dan A. Smale

**Affiliations:** ^1^ Marine Biological Association of the United Kingdom The Laboratory Citadel Hill Plymouth UK; ^2^ Ocean and Earth Science National Oceanography Centre Southampton University of Southampton European Way Southampton UK

**Keywords:** ecology, invasive, management, marine, nonindigenous, *undaria*, Wakame

## Abstract

Marine invasion ecology and management have progressed significantly over the last 30 years although many knowledge gaps and challenges remain. The kelp *Undaria pinnatifida*, or “Wakame,” has a global non‐native range and is considered one of the world's “worst” invasive species. Since its first recorded introduction in 1971, numerous studies have been conducted on its ecology, invasive characteristics, and impacts, yet a general consensus on the best approach to its management has not yet been reached. Here, we synthesize current understanding of this highly invasive species and adopt *Undaria* as a case study to highlight challenges in wider marine invasion ecology and management. Invasive species such as *Undaria* are likely to continue to spread and become conspicuous, prominent components of coastal marine communities. While in many cases, marine invasive species have detectable deleterious impacts on recipient communities, in many others their influence is often limited and location specific. Although not yet conclusive, *Undaria* may cause some ecological impact, but it does not appear to drive ecosystem change in most invaded regions. Targeted management actions have also had minimal success. Further research is needed before well‐considered, evidence‐based management decisions can be made. However, if *Undaria* was to become officially unmanaged in parts of its non‐native range, the presence of a highly productive, habitat former with commercial value and a broad ecological niche, could have significant economic and even environmental benefit. How science and policy reacts to the continued invasion of *Undaria* may influence how similar marine invasive species are handled in the future.

## 1 INTRODUCTION

Globalization is causing an ever‐increasing number of species to be accidentally or intentionally introduced to areas outside of their native range (Perrings, Burgiel, Lonsdale, Mooney, & Williamson, 2010). Estimates include over 50,000 nonindigenous species (NIS) in the USA (Pimentel, Zuniga, & Morrison, 2005) and over 11,000 in Europe (DAISIE, 2009). This prolific exchange of species, coupled with extinctions and reduced biodiversity driven by anthropogenic environmental change, may be causing a progression toward homogenization of the world's flora and fauna (McKinney & Lockwood, 1999). Those NIS which establish, spread, and proliferate without the direct aid of humans are known as “invasive species” (Richardson, Pysek, & Carlton, 2011). Invasive species are considered one of the major drivers of global biodiversity decline (along with changes in climate, land and seabed use, atmospheric CO_2_ and nitrogen deposition; Sala et al., 2000). Invasive species can also cause major economic loss to a variety of industries, including agriculture, forestry, aquaculture, construction, transport, utilities, and tourism, as well as affecting human health (Williams et al., 2010). There are also significant costs associated with research, management, and control. An estimate of total economic cost considering all of these aspects amounts to $120 billion and £1.7 billion per year in the USA and UK, respectively (Pimentel et al., 2005; Williams et al., 2010).

Due to the inherent connectivity within the marine environment, NIS are particularly prevalent and difficult to manage (Eno, Clark, & Sanderson, 1997; Ruiz, Carlton, Grosholz, & Hines, 1997). In six heavily used ports in the USA, Australia, and New Zealand, a new NIS was estimated to establish every 85 weeks, with the fastest rate of introduction every 32 weeks in San Francisco Bay (Hewitt, 2003). Over 250 marine NIS have been identified in Australia (Hewitt, 2003), 150 in New Zealand (Cranfield et al., 1998), 90 in the UK (Minchin, Cook, & Clark, 2013), and over 200 in San Francisco Bay (USA) alone (Cohen & Carlton, 1998). The major vector of introduction is commercial shipping, followed by aquaculture, canals, and aquarium trade (Molnar, Gamboa, Revenga, & Spalding, 2008). Controls on introduction vectors are logistically the most efficient point to inhibit NIS establishment (Bax et al., 2001). However, due to the international, commercial, and public nature of vectors, introductions are unlikely to be completely contained (Hulme, 2006). Once introduced, rapid‐response management may allow eradication at a relatively low control cost (Anderson, 2005; Beric & MacIsaac, 2015), but early recognition of a marine NIS before it establishes is also problematic. Many species have microscopic life stages and are found in inconspicuous and often inaccessible habitats. The incomplete taxonomy and historical records that are apparent for many marine families means that once recognized newly identified species will often be cryptogenic. It can often take considerable time for accurate identification and status of a newly identified species to be determined, requiring a wide range of genetic, ecological, and biochemical techniques, further delaying potential rapid‐response management.

Identifying specific characteristics that predispose a species to being invasive is challenging. Invasive species are generally considered to have high phenotypic or genetic plasticity and a broad ecological niche in order to survive introduction, establishment, and spread in a non‐native range (Kolar & Lodge, 2001; Newsome & Noble, 1986; Williamson & Fitter, 1996; Zenni, Lamy, Lamarque, & Port, 2014). They are often described to have opportunistic life histories, including high fecundity, growth rate, and recruitment; however, there are also successful invasive species with more competitive life‐history traits (Duyck, David, & Quilici, 2007; Valentine, Magierowski, & Johnson, 2007). The probability of invasion increases with the number of individuals released or reproducing, the number of introduction events, and proximity to existing populations (Kolar & Lodge, 2001; Lockwood, Cassey, & Blackburn, 2005). Resource availability, such as light, food, and physical space, is also a key factor which can influence the vulnerability of a recipient community to invasion (Levine & D'Antonio, 1999; Stachowicz, Fried, Osman, & Whitlatch, 2002).

Quantifying the ecological impacts of an invasive species is also complex. Differences in recipient communities, resource availability, environmental abiotic factors, and attributes of the invasive species itself can all create site‐specific impacts. Factors such as abundance and geographical range of the invasive species may influence impacts in all cases (Parker et al., 1999), while other factors such as morphological, behavioral, or even chemical characteristics of the invasive species are more species specific (Thomsen, Olden, Wernberg, Griffin, & Silliman, 2011).

**Figure 1:**
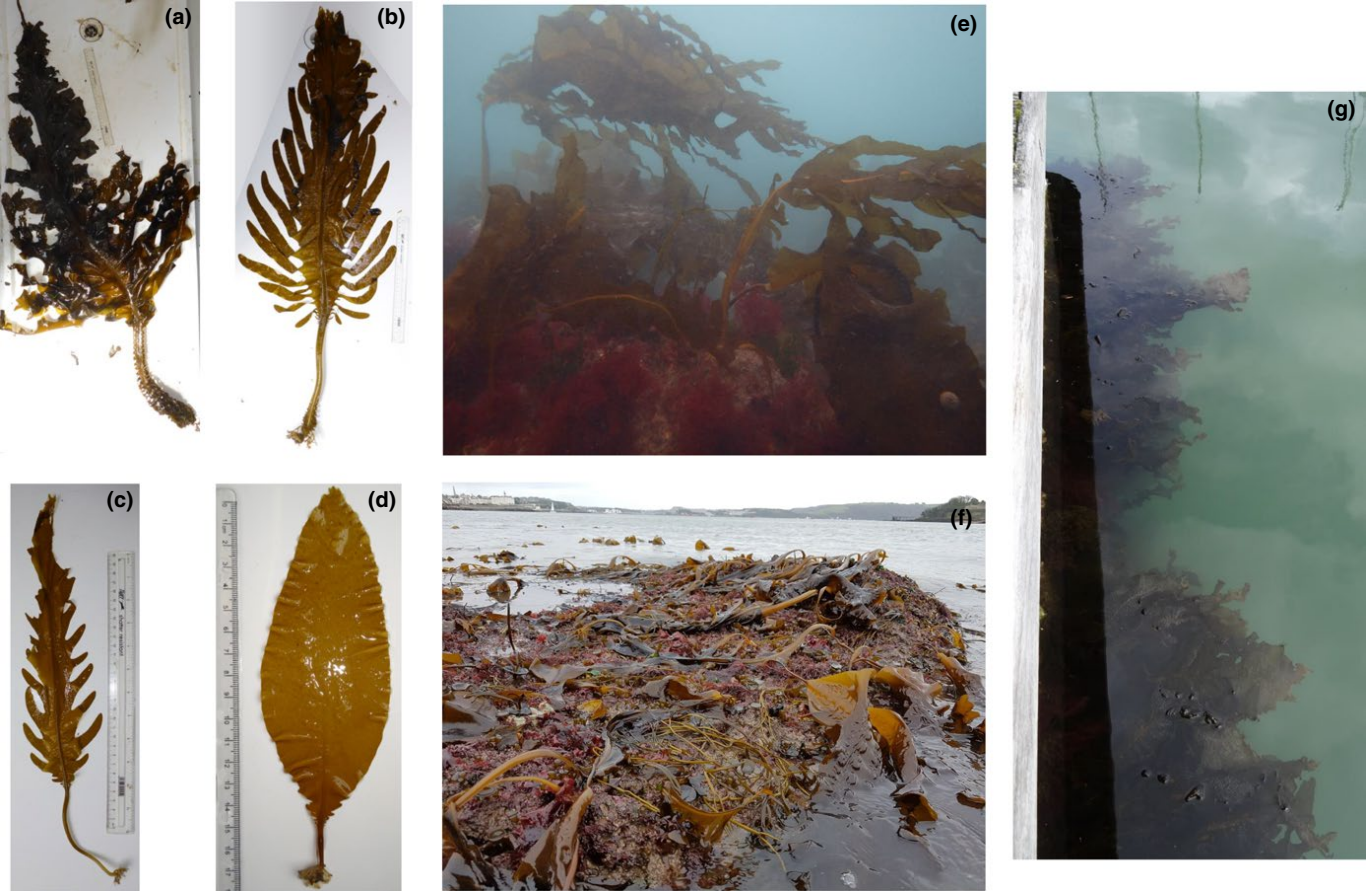
Different developmental stages of *Undaria pinnatifida* sporophytes (a–d). *Undaria pinnatifida* can be found growing in the subtidal and intertidal, as well as on natural and artificial substrates (e‐g)

**Figure 2:**
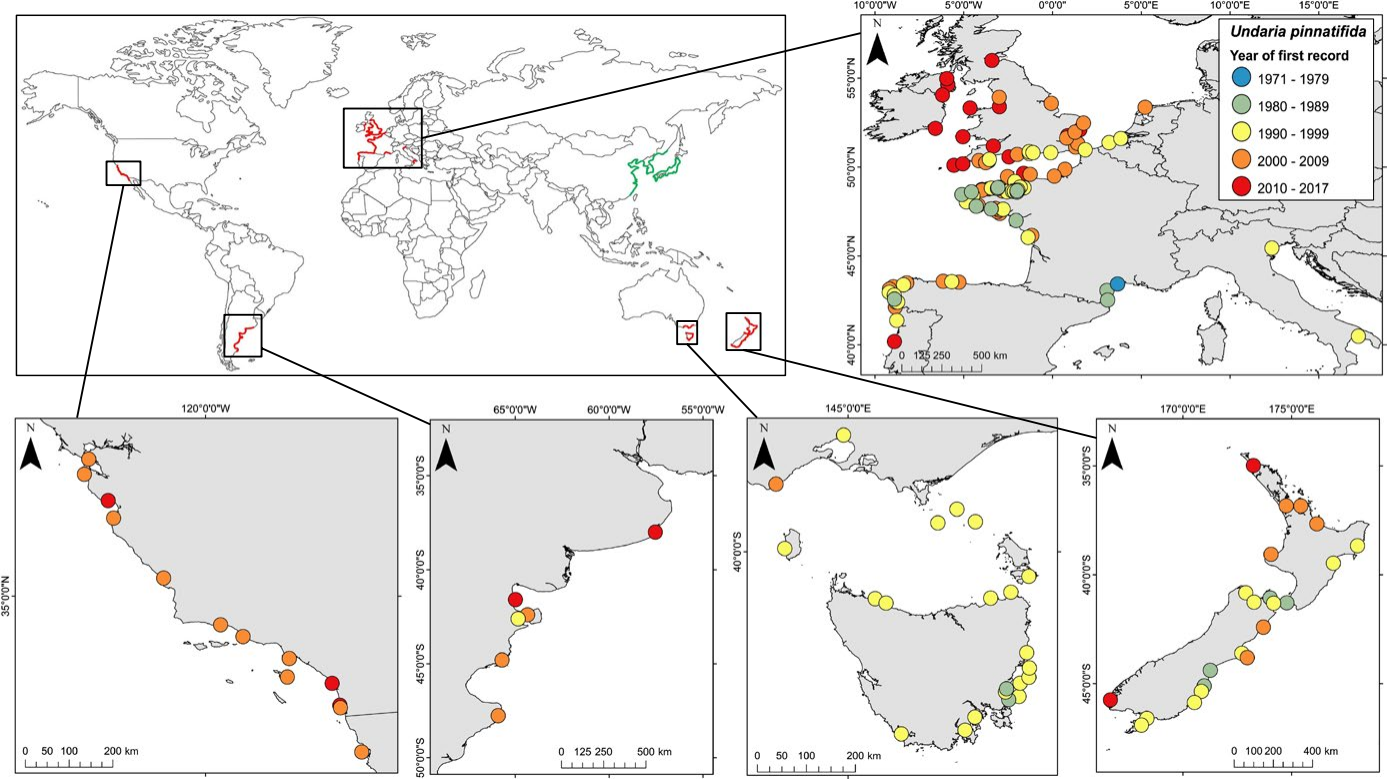
Approximate distribution of *Undaria pinnatifida*. Global map: Green = native range, red = non‐native range. Regional maps: Each point represents a distinct location but does not indicate precise position or entire extent. See Table S1 for more information and references

Invasive marine macroalgae (seaweeds) may function as ecosystem engineers that are able to modify the environment and alter recipient communities and, as such, have the potential to cause significant ecological and socioeconomic impacts (Dijkstra et al., 2017; Thomsen, Wernberg, Tuya, & Silliman, 2009; Williams & Smith, 2007). Overall, there are thought to be approximately 350 different seaweed NIS accounting for around 20%–30% of all marine NIS (Schaffelke & Hewitt, 2007; Thomsen, Wernberg, South, & Schiel, 2016). The cold‐temperate kelp *Undaria pinnatifida* (Figure 1) is one of only two seaweeds (along with *Caulerpa taxifolia*) included in the Invasive Species Specialist Group list of the 100 most invasive species of the world (Lowe, Browne, Boudjekas, & De Poorter, 2000). Native to cold‐temperate areas of the northwest Pacific (the coastlines of Japan, Korea, Russia, and China), the adventive kelp *Undaria pinnatifida* (Harvey) Suringar, 1873 (Phaecophycae, Laminariales), or “Wakame,” has a worldwide non‐native range (Figure 2). First identified as an invasive species on the Mediterranean coast of France in the 1970s (Perez, Lee, & Juge, 1981), *Undaria pinnatifida* (hereafter referred to as *Undaria*) is now established on the coastlines of 13 countries across four continents (James, Kibele, & Shears, 2015). The design of efficient and effective NIS management requires a clear understanding of a species physiology, invasion dynamics, and ecological impacts. Due to its global distribution and status as an invasive species for over 30 years, *Undaria* is a useful case study to highlight both successes and failures in our handling and understanding of marine NIS.

## 2 UNDARIA PINNATIFIDA

### 2.1 Biology, physiology and native ecology

In its native northeast Asia, *Undaria* is a winter annual species that inhabits rocky substrates from the low intertidal to 18 m depth, and is widespread at depths of 1–3 m (Koh & Shin, 1990; Saito, 1975; Skriptsova, Khomenko, & Isakov, 2004). It is also a major species for seaweed mariculture in China, Japan, and Korea (Yamanaka & Akiyama, 1993), with total world yield in 2013 exceeding 2 million tonnes fresh weight (FAO FishStat). Sporophytes can grow up to 1–1.7 cm per day, reach 1.3–2 m in length, and have a maximum life span of around 6–8 months (Castric‐Fey, Beaupoil, Bouchain, Pradier, & L'Hardy‐Halos, 1999; Choi, Kim, Lee, & Nam, 2007; Dean & Hurd, 2007). They form large divided pinnate fronds and distinctive ruffled reproductive sporophylls (Figure 1). As with all kelps, *Undaria* has a heteromorphic life cycle, with large macroscopic diploid sporophytes that produce microscopic zoospores from reproductive sporophylls. The spores develop into microscopic dioecious haploid gametophytes, which, on maturation, produce motile sperm that fertilize the sessile egg and a new sporophyte will start to grow *in situ* of the female gametophyte (Dayton, 1985). Sporophylls develop over several months and mature sequentially from the base upwards (Saito, 1975; Schaffelke, Campbell, & Hewitt, 2005). Zoospores are released over approximately 20–40 days at densities of 0.13 × 10^5^–12 × 10^5^ spores per cm^2^ of sporophyll per hour, amounting to 1 × 10^8^–7 × 10^8^ spores over the lifetime of a sporophyte (Primo, Hewitt, & Campbell, 2010; Saito, 1975; Schaffelke et al., 2005; Schiel & Thompson, 2012). Once released, spores typically move at around 0.13–0.33 mm/s for 5–6 hr, but may remain motile for up to 3 days. Fixing ability starts to be reduced within a few hours, although viability can last over 10 days (Forrest, Brown, Taylor, Hurd, & Hay, 2000; Hay & Luckens, 1987; Saito, 1975; Suto, 1952). Due to the low motility and vitality of the zoospores, settlement is strongly correlated with distance from mature sporophytes, and dispersal may be limited to as little as 0.2–10 m from a spore release point (Forrest et al., 2000; Schiel & Thompson, 2012; Suto, 1952). Larger dispersal distances are thought to be facilitated by the drifting of entire sporophytes, which may remain viable for much longer periods. Overall, it has been estimated that maximum spore‐mediated dispersal rates for populations are in the order of 10–200 m/year, while sporophyte drift may allow maximum dispersal rates of 1–10 km/year (Forrest et al., 2000; Russell, Hepburn, Hurd, & Stuart, 2008; Sliwa, Johnson, & Hewitt, 2006).

**Figure 3:**
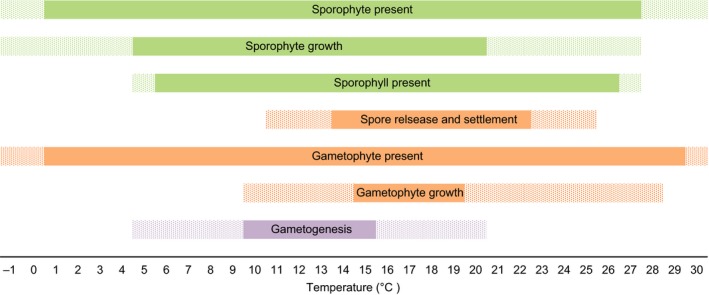
Thermal tolerances of the different life stages of *Undaria pinnatifida*. Lighter colors = life stage possible but may be limited. See in text for references

In most of its native range, *Undaria* sporophyte recruitment occurs in winter, becomes reproductive in spring, and goes through widespread senescence during summer, leaving only the microscopic gametophyte life stages which persist through autumn (Koh & Shin, 1990; Saito, 1975). Temperature is the key environmental factor which determines this annual population dynamic (Figure 3; Saito, 1975). *Undaria* 's native range has average monthly sea‐surface temperatures from −0.6 to 16.8°C in the coldest months, and 23–29.5°C in the warmest months (Dellatorre, Amoroso, Saravia, & Orensanz, 2014; James & Shears, 2016b; Skriptsova et al., 2004; Watanabe, Nishihara, Tokunaga, & Terada, 2014). The ability to tolerate this large annual range is due to the survival of microscopic gametophyte and sporophyte stages which can persist at temperatures between −1 and 30°C (Morita, Kurashima, & Maegawa, 2003a; Saito, 1975). Sporophyte growth has a slightly more restricted temperature range of 0–27°C; optimum growth rate is site‐specific, however, which tends to fall within 5–20°C, and senescence may be induced by exposure to temperatures at or above 24°C (Bollen, Pilditch, Battershill, & Bischof, 2016; Henkel & Hofmann, 2008; James & Shears, 2016a; Morita, Kurashima, & Maegawa, 2003b; Saito, 1975; Skriptsova et al., 2004). The reproductive sporophylls can be present between 5 and 27°C, and when mature, spore release and settlement occur between approximately 11–25°C (James & Shears, 2016b; Saito, 1975; Skriptsova et al., 2004; Thornber, Kinlnan, Graham, & Stachowicz, 2004). Although sporophytes may develop 15–20 days after spore settlement, under certain temperature, light, or competitive regimes, gametophytes may grow vegetatively and remain viable for up to 2 years, thus creating an expanding seed bank from previous generations in the understory (Choi, Young, Soon, Eun, & Ki, 2005; Pang & Wu, 1996; Thornber et al., 2004). The remaining life stages are the most temperature specific and therefore drive the strict annual life cycle in its native range (Figure 3). Gametophyte growth is optimum between 15 and 20°C, while gametogenesis and fertilization is optimum between 10 and 15°C (Henkel & Hofmann, 2008; Morita et al., 2003a; Saito, 1975).

Although less defined than the influence of temperature, many abiotic factors can affect the growth and distribution of *Undaria*, including salinity, light, day length, nutrients, and wave exposure. *Undaria* is predominantly found in fully saline conditions, with mean salinities below 27 psu generally limiting its range (Floc'h, Pajot, & Wallentinus, 1991; Saito, 1975; Watanabe et al., 2014). However, laboratory‐based experiments have shown that zoospore attachment may occur at salinities as low as 19 psu, while gametophytes and sporophytes may survive at salinities as low as 6 psu (although below 16 psu sporophytes may start to become damaged) (Bollen et al., 2016; Peteiro & Sanchez, 2012; Saito, 1975). *Undaria* is viable over a wide range of light regimes; however, changes in irradiance and day length will influence the rate of recruitment, growth, and photosynthesis in both gametophyte and sporophyte stages (Baez et al., 2010; Choi et al., 2005; Morelissen, Dudley, Geange, & Phillips, 2013; Pang & Luning, 2004). Although seasonal and site‐specific, optimal growth occurs around 40–120 μmol m^−2^ s^−1^, light saturation point for photosynthesis (*I*_*k*_) can be reached around 100–500 μmol m^−2^ s^−1^, while the light compensation point (*I*_*c*_; when no net photosynthesis occurs) may be reached between 17 and <5 μmol m^−2^ s^−1^ (Campbell, Bite, & Burridge, 1999; Matsuyama, 1983; Morelissen et al., 2013; Saito, 1975; Watanabe et al., 2014). Although requiring irradiance above approximately 3 μmol m^−2 ^s^−1^ for growth and maturation (Saito, 1975), the gametophyte is able to survive in complete darkness, in a latent phase, for at least 7 months (Kim & Nam, 1997); while zoospore settlement may not be affected by light regime at all (Morelissen et al., 2013).

When compared to perennial or summer annual Laminarians, *Undaria* has a comparatively low rate of nutrient uptake and nitrate storage, and therefore a close association between seawater and tissue nitrate (Dean & Hurd, 2007). This means that growth of sporophyte and gametophyte stages is positively related to nutrient concentration (Dean & Hurd, 2007; Gao, Endo, Taniguchi, & Agatsuma, 2013; Morelissen et al., 2013; Pang & Wu, 1996). Zoospore settlement, however, is not considered to be influenced by nutrient concentration and therefore any inhibition of recruitment by nutrient limitation would occur at the gametophyte or sporophyte stage (Morelissen et al., 2013). Increased water motion can enhance nutrient uptake in kelps (Gerard, 1982), which is highlighted by rope‐based mariculture of *Undaria* being more efficient in moderately exposed sites with water velocities of up to 15–30 cm/s when compared to sheltered sites of 5–12 cm/s (Nanba et al., 2011; Peteiro & Freire, 2011; Peteiro, Sanchez, & Martinez, 2016). Within natural environments, *Undaria* is found at highest abundance in moderately sheltered to moderately exposed open coasts or bays near the open sea (Floc'h, Pajot, & Mouret, 1996; Russell et al., 2008; Saito, 1975). Due to the thin fragile nature of the sporophyte frond, *Undaria* is limited in highly exposed shores (Choi et al., 2007), although can still be found in low intertidal pools or lower subtidal areas, which have more shelter from wave action at exposed sites (Russell et al., 2008). Periods of low water motion are needed for high natural recruitment, with spore adhesion optimal at water velocities of 3 cm/s (Arakawa & Morinaga, 1994). Under certain conditions, spores may completely fail to adhere at flows ≥14 cm/s (Saito, 1975), however, in some cases no inhibition of adhesion rate may occur until flow rates reach over 16 cm/s, and spores may still adhere, albeit at a greatly reduced rate, at flows over 25 cm/s (Arakawa & Morinaga, 1994; Pang & Shan, 2008).

Overall, *Undaria* has a high growth rate, large reproductive output, high phenotypic plasticity, and a relatively wide physiological niche. These factors are often considered characteristic of successful invasive species (Newsome & Noble, 1986; Williamson & Fitter, 1996). On the other hand, *Undaria* exhibits low natural dispersal ability, and its ecophysiological niche is not as broad as some other highly invasive marine macroalgae (Nyberg & Wallentinus, 2005). As such, it could be thought of as a low risk for widespread colonization; however, its invasion history demonstrates it to be a very successful invader.

### 2.2 Invasive characteristics

The primary vectors of introduction and long distance dispersion of *Undaria* were via fouling on the hulls of commercial vessels (Forrest et al., 2000; Hay, 1990; Silva, Woodfield, Cohen, Harris, & Goddard, 2002), and accidental import with shellfish (Floc'h et al., 1991; Perez et al., 1981). *Undaria* was also intentionally introduced for cultivation into Brittany (France) in 1981 (Perez et al., 1981). As with most marine NIS, the initial introductions of *Undaria* therefore all occurred onto artificial substrates within anthropogenic habitats such as harbors, marinas, canals, or modified embayments (e.g., Cremades, Freire, & Peteiro, 2006; Fletcher & Farrell, 1999; Floc'h et al., 1991; Hay & Luckens, 1987; Silva et al., 2002; Zabin, Ashton, Brown, & Ruiz, 2009). Once established, widespread range expansion has been facilitated by human‐mediated transport to other anthropogenic habitats, largely from fouling on commercial and recreational vessels (Dellatorre et al., 2014; Fletcher & Farrell, 1999; Hay, 1990; Kaplanis, Harris, & Smith, 2016; Minchin & Nunn, 2014; Russell et al., 2008; Zabin et al., 2009). Once established in these anthropogenic or modified environments, *Undaria* can spread into natural habitats. Due to its requirement for attachment on hard substrates, it is predominantly found invading rocky reefs; however, it can also be found more rarely to invade sea grass beds and mixed sediment communities (Farrell & Fletcher, 2006; Floc'h et al., 1996; James, Middleton, Middleton, & Shears, 2014; Russell et al., 2008). In many parts of its non‐native range, *Undaria* populations have expanded and, under certain conditions, can make up a significant proportion of canopy‐forming seaweeds. *Undaria'*s dominance is normally seasonal, spatially variable and mostly occurs on artificial substrates in anthropogenic habitats (Castric‐Fey, Girard, & Lhardyhalos, 1993; Curiel, Guidetti, Bellemo, Scattolin, & Marzocchi, 2001; Fletcher & Farrell, 1999; Heiser, Hall‐Spencer, & Hiscock, 2014; James & Shears, 2016a). It can, however, also be found as one of the dominant canopy‐forming seaweeds in natural habitats under certain competitive or environmental settings (Casas, Scrosati, & Piriz, 2004; Heiser et al., 2014; Raffo, Eyras, & Iribarne, 2009; Thompson & Schiel, 2012; Valentine & Johnson, 2003).

Due to the low natural dispersion rates of *Undaria*, local spread of populations tends to occur in a step‐wise manner (Fletcher & Farrell, 1999). The rate of localized natural spread is therefore far lower than human‐mediated spread, with some populations having minimal range expansion for many years following their initial introduction. For example, in the UK it took over 7 years for *Undaria* to colonize a shoreline 200 m away from an established marina population (Farrell & Fletcher, 2006); in the USA, many marina populations remain localized following introductions over 10 years ago (Kaplanis et al., 2016); while in France, it took 10 years for *Undaria* to be found outside of the enclosed lagoon to which it was first introduced (Floc'h et al., 1991). In New Zealand, population expansion seems to be dependent on the area in which it is found. In Timaru Harbour, *Undaria* has extended less than 1 km from the harbor in over 20 years (Russell et al., 2008), in Marlborough Sound, the range of *Undaria* has expanded by hundreds of meters a year (Forrest et al., 2000), and in Moeraki Harbour, expansion was around 1 km per year, while at Otago Harbour, *Undaria* spread around 2 km per year along adjacent exposed coastlines outside the harbor (Russell et al., 2008). Considerably faster rates of spread have also been recorded in areas of Argentina and Australia. Within the San Jose Gulf (Argentina), only 4 years after its introduction, *Undaria* had spread across approximately 100 km of coastline (Dellatorre et al., 2014), and in certain parts of Tasmania, local spread has been estimated to reach up to 10 km per year (Hewitt et al., 2005). Although the rate of range expansion is variable and site‐specific, *Undaria* seems able to spread and proliferate without the direct aid of humans in all of its non‐native range.

As previously discussed, temperature is the key environmental factor which determines the population dynamics of *Undaria* (Saito, 1975). Many parts of *Undaria*'s non‐native range have smaller annual temperature variation than the majority of its native range, meaning thermal cues for its annual life history are lost and some macroscopic sporophytes can be present throughout the year (James et al., 2015; and references therein). Using both *in situ* and satellite‐based temperature measures, it was estimated that where maximum summer sea‐surface temperatures are less than or equal to 19.4°C *Undaria*, sporophytes would be predicted to be present year round, whereas where temperature maxima is greater than or equal to 20.6°C, an annual phenology could be expected (James et al., 2015).

Due to *Undaria* sporophytes living approximately 6–8 months, a recruitment period of four or more months, or multiple recruitment pulses per year could result in the year round presence of sporophytes (James et al., 2015). In Santa Barbara (California, USA) where average sea‐surface temperatures range from approximately 12–19°C, the presence and growth of sporophytes occur year round. There are two recruitment pulses, with a smaller autumn pulse at temperatures from 17 to 21°C, and a larger winter recruitment when temperatures are 12–17°C (Thornber et al., 2004). In this location, recruitment seems to be triggered by a fall in temperature below 15°C, with recruitment occurring around 8 weeks later (Thornber et al., 2004). A similar biannual recruitment has been recorded in New Zealand, with pulses in the autumn and spring (Hay & Villouta, 1993; Thompson & Schiel, 2012). In some areas, such as Brittany (France) and Patagonia (Argentina), sea‐surface temperatures reach over 15°C for only 3–4 months of the year. In these locations, although there are still seasonal pulses, some recruitment occurs year round (Casas, Piriz, & Parodi, 2008; Castric‐Fey et al., 1999; Martin & Bastida, 2008). The ability for *Undaria* to become one of the dominant canopy‐forming seaweeds and have a year round occurrence in parts of its non‐native range, suggests that it could have significant ecological impacts on the recipient communities to which it invades.

### 2.3 Ecological impacts

Surveys examining the distribution of *Undaria* within mixed seaweed assemblages have identified that it occurs more commonly or is found in higher abundance, where there is a lower density of native canopy species (e.g., Castric‐Fey et al., 1993; Cremades et al., 2006; Russell et al., 2008; Heiser et al., 2014; De Leij, Epstein, Brown, & Smale, 2017; Table 1). Due to the lack of pre‐invasion data, it could be argued that *Undaria* may have been the cause of this reduced native canopy. However, results indicate that *Undaria* is occupying substrates, depth ranges, or anthropogenically stressed habitats where native canopy‐forming seaweeds are limited (e.g., Castric‐Fey et al., 1993; Cremades et al., 2006; Russell et al., 2008; James & Shears, 2016b; Table 1). This is supported by an investigation where data on native kelp abundance were available before the *Undaria* invasion. This before‐after control‐impact (BACI) study showed that the introduction of *Undaria* led to no significant change in the abundance of native kelp species over 3 years (Forrest & Taylor, 2002).

**Table 1:**
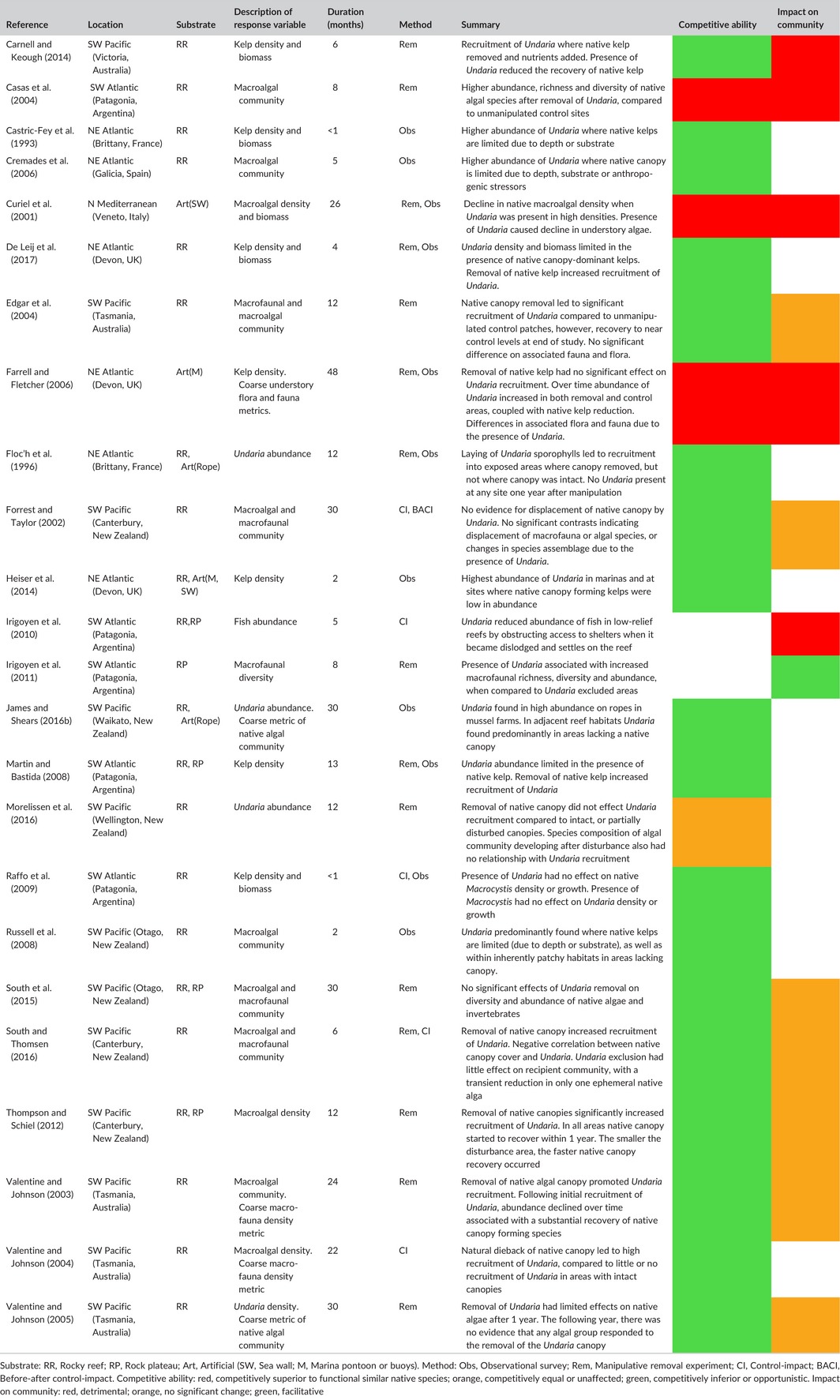
Summary of studies on *Undaria pinnatifida* for which inference could be made to its competitive ability with functionally similar species and its impact on recipient communities

In its native Japan and Korea, *Undaria* can act as a pioneer species and is part of a natural successive colonization process (Agatsuma, Matsuyama, Nakata, Kawai, & Nishikawa, 1997; Kim et al., 2016). Where it has invaded, this pioneer‐like trait is indicated by ecosystem stress or disturbance being key to *Undaria*'s recruitment into mixed canopy assemblages (Table 1). In some cases, stress from eutrophic conditions has been shown to promote *Undaria* recruitment (Carnell & Keough, 2014; Curiel et al., 2001), while canopy disturbance is often a critical factor (De Leij et al., 2017; Edgar, Barrett, Morton, & Samson, 2004; Floc'h et al., 1996; Martin & Bastida, 2008; South & Thomsen, 2016; Thompson & Schiel, 2012; Valentine & Johnson, 2004). Experimental clearance of native kelp species within intertidal and subtidal environments in Australia and New Zealand caused *Undaria* to recruit into manipulated patches, while the following year *Undaria* declined and the native seaweeds started to recover (Thompson & Schiel, 2012; Valentine & Johnson, 2003).

Comparative studies have shown that *Undaria* harbors a distinct and reduced epifaunal and epifloral community when directly compared to native kelp species (Arnold, Teagle, Brown, & Smale, 2016; Raffo et al., 2009). However, as evidence suggests that *Undaria* is not able to displace native kelps, this does not indicate ecological impact in itself. Community‐wide impact studies suggest that the influence of *Undraia* is context‐specific (Table 1). In anthropogenic habitats, *Undaria* may cause a decline in density and diversity of native understory and canopy flora and fauna (Curiel et al., 2001; Farrell & Fletcher, 2006). On natural rocky substrates in Patagonia, there is some evidence that *Undaria* can cause a reduction in diversity and richness of native macroalgae (Casas et al., 2004) and reduce fish abundance (Irigoyen, Eyras, & Parma, 2010), although this may be highly site‐specific. Intertidal studies in New Zealand and Australia have described *Undaria'*s impacts on native biodiversity as transient (Table 1). For example, a two‐and‐half‐year study within intertidal reef habitats in New Zealand repeatedly removed *Undaria* from experimental patches. Measurement of various faunal and floral community indicators showed no long‐term effect of the presence of *Undaria* when compared to control sites (South et al., 2015). A similar result was found in a 3 year BACI study of an *Undaria* invasion into a sheltered embayment of New Zealand, with no evidence of significant ecological impacts on either macroalgae or sessile invertebrates (Forrest & Taylor, 2002).

The distribution, ecological impact, and invasion dynamics of *Undaria* seem to indicate that it is predominantly acting as a passenger of ecosystem change – filling an empty niche or benefiting from resource availability which is temporarily released by ecosystem stress and having a limited impact on recipient communities (Bauer, 2012; Didham, Tylianakis, Hutchison, Ewers, & Gemmell, 2005; MacDougall & Turkington, 2005). There is, however, some evidence that *Undaria* may be driving ecosystem change in certain environments. In a study by Carnell and Keough (2014), *Undaria* required native canopy disturbance to recruit and grow in high abundance; however, under nutrient enhancement, the presence of *Undaria* seemed to limit the recovery of native canopies. In other examples, the native canopy has not inhibited *Undaria* recruitment (Farrell & Fletcher, 2006; Morelissen, Dudley, & Phillips, 2016), and removal or die back of *Undaria* has led to recovery of native macroalgae (Casas et al., 2004; Curiel et al., 2001).

One way in which *Undaria* may be able to drive ecosystem change in the long term is due to its year round presence in some of its non‐native range (Casas et al., 2008; Fletcher & Farrell, 1999; Hay & Villouta, 1993; James & Shears, 2016b). Many larger native canopy‐forming seaweeds are perennial, living up to 10 years, with seasonal growth, reproductive, and senescence stages. If *Undaria* is able to recruit in multiple pulses throughout the year onto available substrate left open by the natural die back of native species, it may be able to slowly monopolize space, increasing in density and excluding native seaweeds. Due to the long life time of some native species, significant increases in the density and distribution of *Undaria* may not be seen for many decades in the absence of wider ecosystem disturbance. Long‐term monitoring and manipulations of *Undaria* invaded communities would be needed in order to demonstrate the potential of this interaction.

It has been suggested that *Undaria* could have facilitative impacts within certain invaded communities, by proving trophic or habitat subsidy (Cecere, Petrocelli, & Saracino, 2000; Irigoyen, Trobbiani, Sgarlatta, & Raffo, 2011; Jimenez et al., 2015; Suarez‐Jimenez et al., 2017). For example, in a low complexity limestone plateau, benthic macrofaunal richness and diversity was higher where *Undaria* was present (Irigoyen et al., 2011). Similarly, within a highly polluted and low diversity enclosed basin of the Ionian Sea the presence of *Undaria* was observed to have a positive ecological function, by increasing benthic primary production and providing food and biogenic habitat for other organisms (Cecere et al., 2000). Further research is needed to better elucidate the net impact (i.e., negative and facilitative) of *Undaria* across a range of invaded ecosystems. To date, the majority of studies have been carried out in the southwest Pacific, yet current evidence suggests that *Undaria* impacts are context‐specific. A key knowledge gap relates to the impacts of *Undaria* in other invaded regions, such as the northwest Atlantic and northeast Pacific. Future research should also include an emphasis on manipulative and BACI studies, as well as long‐term monitoring activities and comparative work across large spatial scales, in order to causally determine the effects of *Undaria* within invaded ecosystems.

### 2.4 Management

Management frameworks designed to control the abundance and spread of *Undaria* could only be found for two of the countries to which it has been introduced (Table 2). These are largely generic, with measures applicable to wider NIS introductions. For example, the key measures recommended for managing *Undaria* in New Zealand include surveillance and response to new infestations in high‐value areas, vector monitoring and control, prohibition of intentional release, controls on ballast water discharge, improved research, education, and public awareness (Sinner, Forrest, & Taylor, 2000). Although not necessarily a requirement, none of these measures will reduce localized natural spread or abundance of *Undaria*.

**Table 2:** Status and management of *Undaria pinnatifida* within its non‐native range

Country	First recorded	Population status	Dedicated management plan	Summary of known management	Management aim	References
France	1971	Common in natural and anthropogenic habitats across current range. Active mariculture	None found	Mariculture limited to areas with already developed infrastructure and high *Undaria* abundance. Mariculture under strict control to prevent potential ecological impacts and further spread.	Inhibit range expansion	Antoine et al. (2012); Castric‐Fey et al. (1993)
New Zealand	1987	Common in natural and anthropogenic habitats across current range. Active mariculture	Sinner et al. (2000)	Surveillance and response to new infestations in high‐value areas, vector monitoring and control, prohibition of intentional release, controls on ballast water discharge, improved research, education, and public awareness	Inhibit range expansion	Russell et al. (2008); James et al. (2014)
Spain	1988	Common in natural and anthropogenic habitats across current range. Active mariculture.	None found	*Undaria* not included as an invasive or potentially invasive species within invasive alien species legislation.	Unmanaged	Baez et al. (2010); BOE (2013)
Australia	1988	Common in natural and anthropogenic habitats across current range	NSPMMPI (2015)	Reduce spread to high value areas, possible commercial harvest with tight biosecurity, modify dry‐dock timing to minimize sporophyte development, maintain integrity of native canopy algae, ballast water management, monitoring	Inhibit range expansion	Valentine and Johnson (2004); Primo et al. (2010)
Italy	1992	Largely confined to heavily modified environments and on artificial substrates	None found	None found	None found	Cecere et al. (2000); Curiel et al. (2001)
UK & ROI	1994	Confined to anthropogenic habitats in many locations. Common in natural habitats in parts of the south English and Welsh coast	None found	None found	None found	Heiser et al. (2014); Minchin and Nunn (2014); Wood, Bishop, and Yunnie (2015)
Portugal	1999	Found at only one marina and one natural reef site	None found	None found	None found	Veiga, Torres, Rubal, Troncoso, and Sousa‐Pinto (2014)
Belgium	1999	Uncertain. Likely to be predominantly in ports across current range	None found	None found	None found	Leliaert, Kerckhof, and Coppejans (2000); VLIZ (2011)
Holland	1999	Predominantly in anthropogenic habitats in the Wadden Sea. In natural and anthropogenic habitats in Oosterschelde	None found	Recommendations for a national coordinated management plan	Inhibit range expansion	Gittenberger and Stegenga (2013); Verbrugge et al. (2015)
USA	2000	Largely confined to anthropogenic habitats (Only two records on natural reef in 2001)	None found	Academic and citizen science led research and removal from marinas in California	Inhibit range expansion	Kaplanis et al. (2016)
Argentina	2000	Common in natural and anthropogenic habitats across current range	None found	Manual removal of macroscopic sporophytes and a regular monitoring program to track and eventually prevent its dispersal within one province	Inhibit range expansion	Dellatorre et al. (2014)
Mexico	2003	Isolated island population on natural reef	None found	None found	None found	Aguilar‐Rosas, Aguilar‐Rosas, Avila‐Serrano, and Marcos‐Ramirez (2004)

Eradication using heat treatment has been successful where an isolated population occurred on a wrecked trawler in the Chatham Islands, New Zealand (Wotton, O'Brien, Stuart, & Fergus, 2004). Removal of all sporophytes over a 15‐month period led to the long‐term eradication of *Undaria* from the site and inhibited its spread to natural substrates. Even at this small scale, eradication cost around $0.4 million (NZD). Eradication from longer established populations in natural environments has not yet been successful. A management trial in Tasmania removed *Undaria* monthly from a 800 m^2^ area of rocky reef. Although there was a significant reduction in sporophyte abundance, eradication was not achieved, with sporophytes present at each subsequent visit (Hewitt et al., 2005). Experimental manipulations carried out in New Zealand and Italy, whereby small (0.5 m^2^) areas of *Undaria* dominated rocky substrate were scraped clean, also saw fresh recruitment within 1 year (Curiel et al., 2001; Thompson & Schiel, 2012).

As previously discussed, many studies have shown that *Undaria* requires a level of ecosystem stress or disturbance to recruit and spread in mixed seaweed canopies. Reducing, mitigating, or preventing anthropogenic disturbance to native canopies has therefore been suggested as a management option to prevent the spread, and limit the abundance of *Undaria* (Valentine & Johnson, 2003). However, where *Undaria* has already established at high densities, or if it is acting as a “backseat driver” – suppressing native species once recruited (Bauer, 2012), maintaining native canopies alone is unlikely to be effective (Valentine & Johnson, 2003).

The management options available to directly target the local spread and abundance of *Undaria* are unclear. Where *Undaria* can be found in multiple locations and at high abundance within natural environments, it seems unlikely that eradication would be feasible. This is generally accepted by environmental managers, with widespread eradication of *Undaria* not currently being considered in any country to which it has been introduced (Table 2). Due to the importance of artificial or anthropogenic environments in the establishment of *Undaria* and its relatively low natural dispersal rates, control of new or isolated populations should be plausible. Monitoring of harbors, marinas, ports, high‐value natural areas and natural boundaries, with rapid‐response eradication to any new sightings, could greatly reduce wide‐scale spread of *Undaria*, and therefore, the ecological impacts it may have on natural habitats (Forrest, Gardner, & Taylor, 2009). In New Zealand, *Undaria* is currently absent from the west coast of the South Island, and large areas of the North Island's west coast. In April 2010, a mature sporophyte was found within Sunday Cove, Fiordland World Heritage Area, on the west coast of the South Island (ES, 2016). Since that time, dive‐based surveys and removal of *Undaria* have been carried out every 4–5 weeks at a cost over $1 million (NZD). Six years after the commencement of the program, occasional young individuals are still found; however, it is still the aim of managers to entirely eradicate *Undaria* from the area (ES, 2016).

In many regions where *Undaria* is now accepted (i.e., eradication is no longer being considered), commercial farming and wild harvest are being developed. Mariculture expanded across Brittany, after *Undaria'*s initial introduction in 1981, with nine sites established into the early 1990s (Castric‐Fey et al., 1993). Cultivation and mariculture have also been carried out on the Galician coast of Spain since the late 1990s and are continuing to develop along the North coast (Perez‐Cirera et al., 1997; Peteiro et al., 2016). In 2010, The Ministry for Primary Industries (New Zealand) introduced a revised policy for the commercial use of *Undaria* which approved its wild harvest from artificial substrates or when cast ashore in selected areas. It also approved mariculture in three heavily infested areas, but prohibited harvest from natural substrates unless part of a designated control program (MAF, 2010). The rationale behind the prohibition of harvest from natural substrates was that “it could disturb or remove native canopy species leading to a proliferation of *Undaria*,” while “harvesting when taken as part of a control program is allowed as any risks associated with harvest will be outweighed by reduced *Undaria* in localized areas” (MAF, 2010). It may be possible that one of the remaining options to reduce the abundance and local spread of *Undaria* where eradication is no longer feasible, would be through the legalization of commercial wild harvest from natural substrates. Strict biosecurity would have to be implemented to avoid its spread, and harvesting practices would need to minimize damage to native canopies—such as through a licensing system for hand harvesting only in specific areas. Timings of harvest would also have to be carefully considered, as removal or thinning of the *Undaria* canopy can result in a strong positive response of conspecific recruitment, and increased growth rate of the remaining stock (Gao, Endo, Taniguchi, & Agatsuma, 2014; Thompson & Schiel, 2012). However, removal before maturation could greatly reduce spore and seed‐bank densities, and would perhaps limit the abundance and spread of *Undaria* over time.

Decisions taken by environmental managers on whether to manage *Undaria* within a given jurisdiction should be made on a case‐by‐case basis. Where *Undaria* has recently arrived, or has a restricted range, it is likely that there will be a better chance of successful control or eradication. However, due to the widespread global distribution of *Undaria*, re‐introduction is probable without the implementation of thorough biosecurity. The native community into which *Undaria* is introduced may also strongly influence the decisions of environmental managers. The invasion of *Undaria* is likely to have greater ecological impact in areas where there are no functionally similar native species, whereas, in communities which are dominated by native canopy‐forming macroalgae, *Undaria* may have limited impact on the community as a whole, and act as a passenger of ecosystem change. Economics and the maintenance of ecosystem services will also be factors that influence the decisions made by environmental managers. Although not covered as part of this review, *Undaria* can act as fouling pest to industries such as aquaculture, shipping, and recreational boating (Hay, 1990; James & Shears, 2016a; Minchin & Nunn, 2014; Zabin et al., 2009). The overall economic impacts of this interaction are poorly understood, but as has been noted above, *Undaria* could also have economic benefit through the development of an *Undaria* mariculture industry. Careful consideration and further research is needed on a site‐specific basis. Clearly, the risks, costs, impacts, and benefits of all options, including potential management or eradication and possible acceptance, should be considered when developing management plans for *Undaria*.

## 3 LESSONS LEARNT FOR WIDER MARINE INVASION ECOLOGY

### 3.1 Predicting invaders and reacting to NIS

Although our understanding of marine NIS has greatly increased, *Undaria* is a useful case study to demonstrate that current capacity to predict the invasion dynamics of many marine NIS, and their interactions and impacts within native communities, remains limited. Once introduced, most NIS would not be expected to establish or become invasive (Lodge, 1993; Williamson & Fitter, 1996). Where invasion does occur, the time from initial introduction to when a species becomes invasive is highly variable. In some cases this ``lag time” may last decades, with little‐to‐no proliferation of NIS populations for a considerable time after introduction (Crooks, 2005). This is highlighted by the invasion history of *Undaria*, which has exhibited a wide range of expansion rates following introduction into different regions. Predicting which NIS are likely to become invasive can therefore be challenging. Species traits are often used to predict which NIS may become invasive (Newsome & Noble, 1986; Williamson & Fitter, 1996), although this approach has limitations (Duyck et al., 2007; Kolar & Lodge, 2001; Nyberg & Wallentinus, 2005).

*Undaria* was considered to be an acceptable species for intentional introduction into France for mariculture purposes in 1981 (Perez et al., 1981). A better understanding of a species ecology and physiology is required before intentional introductions are conducted. However, when adventive species arrive unexpectedly, the necessity for rapid‐response management negates this consideration. A failure to react to new introductions could have major consequences. As marine invasive species can cause significant damage to the environment and economy, and due to the complex nature of species invasions, a precautionary principle should be adopted to minimize the rate of any new introductions (Bax, Williamson, Aguero, Gonzalez, & Geeves, 2003; Grosholz, 2002; Molnar et al., 2008).

### 3.2 Ecological impacts

For some marine invasive species, deleterious ecological impacts can be substantial and easy to detect. Introduced voracious predators such as the northern Pacific seastar, *Asterias amurensis*, in Tasmania (Ross, Johnson, & Hewitt, 2003), the Lionfish, *Pterois volitans*, in the tropical Atlantic (Green, Akins, Maljkovi, & Ct, 2012) and the North American mud crab *Rhithropanopeus harrisii* in the Baltic Sea (Jormalainen, Gagnon, Sjroos, & Rothusler, 2016), prey on wide range of native species and proliferate in the absence of native predators. In these examples, clear community‐wide impacts can be identified. Similarly, when invasive species greatly alter nutrient pathways, trophic interactions, or habitat structure, impacts at the community and ecosystem level are easily detectable (Crooks, 2002; Simberloff, 2011). For example, colonial ascidians of the genus *Didemnum* have overgrown large areas of hard substrates, particularly in the Netherlands and USA. These “mats” can greatly alter the physical habitat, cause mortality through smothering of sessile flora and fauna, and have major deleterious impact on wider ecosystem functioning with socioeconomic consequences (Bullard et al., 2007; Gittenberger, 2007). The invasion of *Undaria* highlights that in many other cases, ecological impacts are far harder to quantify and may vary considerably between locations and recipient communities. For these species, justifying costly eradication attempts may be challenging. However, as marine invasive species spread to new regions, decisions will have to be made on potential rapid‐response management before site‐specific impact studies can be carried out.

Invasive species, including *Undaria*, can also have facilitative impacts on the recipient community (Dijkstra et al., 2017; Irigoyen et al., 2011; Rodriguez, 2006). The invasion of bivalve molluscs onto soft sediments, such as *Musculista senhousia* and *Crassostrea gigas*, is a useful example of facilitation by a marine invasive on multiple levels. They provide complex habitats which can greatly increase infaunal and epifaunal abundance, increase organic content in sediment to the benefit of associated organisms, and can act as a trophic subsidy to predatory invertebrate and vertebrate species (Crooks & Khim, 1999; Escapa et al., 2004; Padilla, 2010). In order to understand the overall ecological impact a marine invasive species has on the recipient community, both deleterious and facilitative effects must be considered. Intrinsically, the facilitation of one species is likely to occur at the expense of others, due to changes in competition or predation. In fact for both *Musculista senhousia* and *Crassostrea gigas*, where high densities are found, a reduction in the abundance of functionally similar native species is often recorded (Creese, Hooker, De Luca, & Wharton, 1997; Crooks & Khim, 1999; Padilla, 2010). In many cases, unequivocal evidence of significant ecological impact of an invasive species on recipient communities will be difficult to attain. Prioritization of management actions will be influenced by the perceived impacts of marine invasive species, in terms of their threat to conservation and the maintenance of ecosystem services across different regions, as well as their direct socieoeconomic impacts.

### 3.3 Management

Managing marine NIS is expensive and time‐consuming, while eradication may be impossible once a species is established and widespread (Hulme, 2006). There are examples of successful rapid‐response eradication of invasive species in the marine environment. The seaweed *Caulerpa taxifolia* was first identified in the USA in 2000 (Jousson et al., 2000). A rapid response only 17 days after its first discovery allowed the successful implementation of a 5‐year eradication program using containment and chemical treatment, at a cost of around $7.5 million (USD) (Anderson, 2005). However, as shown by *Undaria*, once a marine NIS is established, proliferation and spread may be inevitable due to the natural or engineered connectivity of many water bodies. As population size increases the costs of control also increase, while attempting eradication of established populations would require significant resources and effort, and may ultimately be unsuccessful (Hobbs & Humphries, 1995). A pertinent example of a marine invasive species where targeted management was deemed to be inappropriate is the macroalgae *Sargassum muticum* or “Japanese wireweed” in Europe. After its introduction into the UK in 1973, *Sargassum* spread across much of Europe's northeast Atlantic and Mediterranean coastlines. A variety of impact studies have been carried out in different parts of its non‐native range with varying results. Some studies found it to alter the recipient community to which it was introduced (Harries, Harrow, Wilson, Mair, & Donnan, 2007; Staehr, Pedersen, Thomsen, Wernberg, & Krause‐Jensen, 2000; Viejo, 1997), however, other long‐term studies recorded limited effects from the invasive species (Olabarria, Rodil, Incera, & Troncoso, 2009; Sanchez & Fernandez, 2005). Although attempts at management were made (Critchley, Farnham, & Morrell, 1986), due to its widespread distribution, uncertainties in the level of its ecological impact, as well as the costs and difficulties in its control, *Sargassum* now has no targeted management across most of Europe.

As with many other invasive species, *Undaria* has a largely opportunistic life strategy, taking advantage of resource availability in order to establish and spread (Gurevitch & Padilla, 2004). These species are sometimes considered “passengers” – promoted and maintained due to the presence of ecosystem stress or disturbance but not in themselves the cause of ecosystem change (MacDougall & Turkington, 2005). A potential management option for these species is not to directly target the species itself, but instead to manage the causes of ecosystem stress or disturbance, with the ultimate aim of restoring, maintaining or even promoting the diversity, integrity, and biotic resistance of recipient communities to invaders. Managing long‐term global‐scale stressors such as climate change will be challenging but crucial given the known interactions between climate and the spread of NIS (Occhipinti‐Ambrogi, 2007). On a local‐to‐regional scale, however, managing stressors such as coastal inputs of sediments and nutrients and physical disturbances from resource extraction, fishing activities, and coastal development may allow some biotic resistance to be maintained. While designing and prioritizing targeted management options for invasive species is of significant importance, especially for those that are considered of high risk or highly damaging, it is also clear that attention should be given to preserving the integrity, diversity, and resistance of native communities through maintaining good overall environmental status. This has been shown for *Undaria*, as its abundance and spread is limited by the presence of diverse, native macroalgae canopies (e.g. Castric‐Fey et al., 1993; De Leij et al., 2017; Russell et al., 2008; Valentine & Johnson, 2003, 2004).

As marine NIS continue to spread and extend their non‐native ranges, decisions will be made on the necessity and feasibility of managing new incursions. Although a precautionary principle should be applied, it is unrealistic to assume that management and control of all species can be achieved due to the widespread establishment of many marine invasive species. Difficult choices will have to be made regarding which species should be targeted, with some potentially becoming an accepted part of the local biota. These decisions must be made on a case‐by‐case basis using the best information available and will depend on a variety of factors including the likely effectiveness, practicality, risk and cost of management options, as well as negative and positive ecological and socioeconomic impacts of a given species.

### 3.4 Accepting NIS

Many NIS have been established in their non‐native range for a considerable time and are now considered part of the natural biota in different regions across the world with major economic benefit and even cultural importance (Davis et al., 2011; Ewel et al., 1999). These species frequently occur in high abundance and over a wide distribution, and could therefore be classed as invasive. Due to the historic nature of species introductions, the widespread acceptance of certain NIS or invasive species is particularly common in the terrestrial environment. The vast majority of the world's agricultural and horticultural species are NIS where they are grown. Many freshwater fish species have also been historically introduced for farming and sports fishing purposes and are treated essentially as part of the natural biota in many regions (Copp et al., 2005; Eustice, 2014; Gozlan, 2008).

In the marine environment, there is a tendency for all NIS to be classed as damaging invasives; however, many species have been established outside their native range for many decades, with little‐to‐no reported impacts. Although further intentional spread may be restricted, few have targeted management plans aiming to reduce their abundance, and are in practice, treated the same as native species. An example of a marine species where perceptions are changing is the Pacific Oyster, *Crassostrea gigas*. The oyster has been intentionally introduced from Asia for farming across the world since the late 1800s. Although initially believed unable to reproduce in the lower sea temperatures around the cold‐temperate Pacific and Atlantic coasts, wild populations have established in most introduced regions. In some cases, this species is considered as a damaging invasive, with management being developed, or enforced to reduce its spread (Guy & Roberts, 2010; NSW, 1994). However, in many parts of the USA and France, where introductions occurred in the 1920s and 1960s, respectively, they are now being seen as part of the natural biota, and are targeted by both wild capture fisheries and aquaculture using seeded bottom culture techniques (Buestel, Ropert, Prou, & Goulletquer, 2009; Cognie, Haure, & Barill, 2006; Feldman, Armstrong, Dumbauld, DeWitt, & Doty, 2000).

Although somewhat contentious, in certain cases invasive species could be considered to have benefits to nature conservation (Schlaepfer, Sax, & Olden, 2011, 2012; Vitule, Freire, Vazquez, Nuez, & Simberloff, 2012). This may occur if the invasive species (i) has considerable facilitative and minimal deleterious impacts on native species; (ii) acts as a catalyst for restoration of native habitats; (iii) functionally replaces a limited or extinct native species; (iv) facilitates a species of high conservation value; or (v) acts as a biocontrol agent (Schlaepfer et al., 2011). These benefits are again more commonly identified in the terrestrial environment due to the historical and often intentional nature of introductions (e.g. Lugo, 2004; Morrison, Reekie, & Jensen, 1998). *Crassostrea gigas* may be another pertinent example relating to the marine environment. In many parts of Europe and America, native oysters have been over harvested and are considered endangered. It has been suggested that the spread of the invasive Pacific Oyster may have conservation benefit, functionally replacing the native species, providing habitat, a trophic subsidy and increased biofiltration, while also providing an exploitable resource, reducing further harvesting pressure on the native homolog (Paalvast, van Wesenbeeck, van der Velde, & de Vries, 2012; Shpigel & Blaylock, 1991).

As previously stated, some marine invasive species, such as voracious predators, or those with perennial life cycles and more competitive life‐history traits, can have major detrimental ecological impact. Many of these species also have minimal facilitative impacts and may lack any societal benefits. These species are unlikely to be accepted and may require prolonged management or control. *Undaria*, however, is a large primary producer, which may provide a trophic and habitat subsidy to native communities within some systems. Although more site‐specific research is needed, in many cases, it has also been recorded as having minimal deleterious impact on native species. There is also commercial potential, with both wild harvest and rope‐based mariculture conducted in parts of *Undaria*'s non‐native range (Castric‐Fey et al., 1993; MAF, 2010; Perez‐Cirera et al., 1997; Peteiro et al., 2016). In areas where likelihood of controlling *Undaria* is low due to widespread established populations, and context‐specific studies show limited ecological impact, it may be that *Undaria* becomes one of few marine invasive species accepted as part of the local biota, with the potential for further development as a commercial resource.

## 4 CONCLUSIONS

There are many challenges facing the future of marine invasion ecology. Total prevention of introductions of new NIS is highly unlikely, while management or eradication is extremely costly and often infeasible. Invasive species are likely to continue their spread and become conspicuous and prominent components of coastal marine communities. In many cases marine invasive species have clearly detectable deleterious impacts on recipient communities; however, in many others their influence is often limited and site‐specific. *Undaria* has now been established for over 40 years in some of its non‐native range. In these areas, rapid response or eradication is no longer an option and the need for any targeted management should be considered. Although not yet conclusive, *Undaria* seems to have minimal ecological impacts in most invaded locations and does not appear to be a “driver” of ecosystem change in most contexts. If this is shown to be the case, it may be more beneficial to target management effort toward the causes of ecosystem stress that reduce native biotic resistance and allow *Undaria* to proliferate, rather than attempting to exclude the species itself. Further research is needed before well‐considered, evidence‐based management decisions can be made on a case‐by‐case basis. However, if *Undaria* was to become officially “unmanaged” in parts of its non‐native range and accepted as a component of the native flora, the presence of a habitat forming, primary producer with a broad ecological niche and potential commercial value, may deliver significant economic and even environmental benefit. How science and policy reacts to the continued spread and proliferation of *Undaria* may influence how similar marine invasive species are handled in the future.

## CONFLICT OF INTEREST

None declared.

## AUTHOR CONTRIBUTIONS

G.E. is the primary author and produced the majority of the content of this review. D.A.S. was involved throughout the process from first draft to final manuscript, including conception, composition, critical review, and final approval for submission.

## References

[ece33430-bib-0001] Agatsuma, Y. , Matsuyama, K. , Nakata, A. , Kawai, T. , & Nishikawa, N. (1997). Marine algal succession on coralline flats after removal of sea urchins in suttsu bay on the Japan sea coast of Hokkaido, Japan. Nippon Susian Gakkaishi, 63(5), 672–680.

[ece33430-bib-0002] Aguilar‐Rosas, R. , Aguilar‐Rosas, L. E. , Avila‐Serrano, G. , & Marcos‐Ramirez, R. (2004). First record of *Undaria pinnatifida* (Harvey) Suringar (laminariales, phaeophyta) on the pacific coast of Mexico. Botanica Marina, 47(3), 255–258.

[ece33430-bib-0003] Anderson, L. W. J. (2005). California's reaction to *Caulerpa taxifolia*: A model for invasive species rapid response. Biological Invasions, 7(6), 1003–1016.

[ece33430-bib-0004] Antoine, L. , Lemoine, M. , Boulben, S. , Kaas, R. , Laurans, M. , Viard, F. , & Potin, P. (2012). Emergence d'une filiere de culture de macro‐algues en bretagne et probleme relatifa une espece non indigene, le wakame (Undaria pinnatifida). Report, IFREMER, 16 pp.

[ece33430-bib-0005] Arakawa, H. , & Morinaga, T. (1994). The rate of brown algal zoospores adhered in the seaweed substrate in relation to gradient of the substrate. Nippon Suisan Gakkaishi, 60(4), 461–466.

[ece33430-bib-0006] Arnold, M. , Teagle, H. , Brown, M. P. , & Smale, D. A. (2016). The structure and diversity of epibiotic assemblages associated with the invasive kelp *Undaria pinnatifida* in comparison to native habitat‐forming macroalgae on a subtidal temperate reef. Biological Invasions, 18(3), 661–676.

[ece33430-bib-0007] Baez, J. C. , Olivero, J. , Peteiro, C. , Ferri‐Yanez, F. , Garcia‐Soto, C. , & Real, R. (2010). Macro environmental modelling of the current distribution of *Undaria pinnatifida* (Laminariales, Ochrophyta) in northern Iberia. Biological Invasions, 12(7), 2131–2139.

[ece33430-bib-0008] Bauer, J. T. (2012). Invasive species: “back‐seat drivers” of ecosystem change? Biological Invasions, 14(7), 1295–1304.

[ece33430-bib-0009] Bax, N. , Carlton, J. T. , Mathews‐Amos, A. , Haedrich, R. L. , Howarth, F. G. , Purcell, J. E. , … Gray, A. (2001). The control of biological invasions in the world's oceans. Conservation Biology, 15(5), 1234–1246.

[ece33430-bib-0010] Bax, N. , Williamson, A. , Aguero, M. , Gonzalez, E. , & Geeves, W. (2003). Marine invasive alien species: A threat to global biodiversity. Marine Policy, 27(4), 313–323.

[ece33430-bib-0011] Beric, B. , & MacIsaac, H. J. (2015). Determinants of rapid response success for alien invasive species in aquatic ecosystems. Biological Invasions, 17(11), 3327–3335.

[ece33430-bib-0012] BOE (2013). Real decreto 630/2013, de 2 de agosto, por el que se regula el catlogo espaol de especies exticas invasoras. Boletn Oficial del Estado No. 185, 56764–56786.

[ece33430-bib-0013] Bollen, M. , Pilditch, C. A. , Battershill, C. N. , & Bischof, K. (2016). Salinity and temperature tolerance of the invasive alga *Undaria pinnatifida* and native New Zealand kelps: Implications for competition. Marine Biology, 163, 194.

[ece33430-bib-0014] Buestel, D. , Ropert, M. , Prou, J. , & Goulletquer, P. (2009). History, status, and future of oyster culture in France. Journal of Shellfish Research, 28(4), 813–820.

[ece33430-bib-0015] Bullard, S. G. , Lambert, G. , Carman, M. R. , Byrnes, J. , Whitlatch, R. B. , Ruiz, G. , … Heinonen, K. (2007). The colonial ascidian Didemnum sp. A: Current distribution, basic biology and potential threat to marine communities of the northeast and west coasts of North America. Journal of Experimental Marine Biology and Ecology, 342(1), 99–108.

[ece33430-bib-0016] Campbell, S. J. , Bite, J. S. , & Burridge, T. R. (1999). Seasonal patterns in the photosynthetic capacity, tissue pigment and nutrient content of different developmental stages of *Undaria pinnatifida* (Phaeophyta: Laminariales) in Port Phillip Bay, South‐Eastern Australia. Botanica Marina, 42(3), 231–241.

[ece33430-bib-0017] Carnell, P. E. , & Keough, M. J. (2014). Spatially variable synergistic effects of disturbance and additional nutrients on kelp recruitment and recovery. Oecologia, 175(1), 409–416. 24604540 10.1007/s00442-014-2907-9

[ece33430-bib-0018] Casas, G. N. , Piriz, M. L. , & Parodi, E. R. (2008). Population features of the invasive kelp *Undaria pinnatifida* (Phaeophyceae: Laminariales) in Nuevo Gulf (Patagonia, Argentina). Journal of the Marine Biological Association of the United Kingdom, 88(1), 21–28.

[ece33430-bib-0019] Casas, G. , Scrosati, R. , & Piriz, M. L. (2004). The invasive kelp *Undaria pinnatifida* (Phaeo‐phyceae, Laminariales) reduces native seaweed diversity in Nuevo Gulf (Patagonia, Argentina). Biological Invasions, 6, 411–416.

[ece33430-bib-0020] Castric‐Fey, A. , Beaupoil, C. , Bouchain, J. , Pradier, E. , & L'Hardy‐Halos, M. T. (1999). The introduced alga *Undaria pinnatifida* (Laminariales, Alariaceae) in the rocky shore ecosystem of the St Malo area: Morphology and growth of the sporophyte. Botanica Marina, 42(1), 71–82.

[ece33430-bib-0021] Castric‐Fey, A. , Girard, A. , & Lhardyhalos, M. T. (1993). The distribution of *Undaria pinnatifida* (Phaeophyceae, Laminariales) on the coast of St. Malo (Brittany, France). Botanica Marina, 36(4), 351–358.

[ece33430-bib-0022] Cecere, E. , Petrocelli, A. , & Saracino, O. D. (2000). *Undaria pinnatifida* (Fucophyceae, Laminariales) spread in the central Mediterranean: its occurrence in the mar piccolo of Taranto (Ionian Sea, southern Italy). Cryptogamie Algologie, 21(3), 305–309.

[ece33430-bib-0023] Choi, H. G. , Kim, Y. S. , Lee, S. J. , & Nam, K. W. (2007). Growth and reproductive patterns of *Undaria pinnatifida* sporophytes in a cultivation farm in Busan, Korea. Journal of Applied Phycology, 19(2), 131–138. 19396348 10.1007/s10811-006-9119-6 PMC2668580

[ece33430-bib-0024] Choi, H. G. , Young, S. K. , Soon, J. L. , Eun, J. P. , & Ki, W. N. (2005). Effects of daylength, irradiance and settlement density on the growth and reproduction of *Undaria pinnatifida* gametophytes. Journal of Applied Phycology, 17(5), 423–430.

[ece33430-bib-0025] Cognie, B. , Haure, J. , & Barill, L. (2006). Spatial distribution in a temperate coastal ecosystem of the wild stock of the farmed oyster *Crassostrea gigas* (Thunberg). Aquaculture, 259(1–4), 249–259.

[ece33430-bib-0026] Cohen, A. N. , & Carlton, J. T. (1998). Accelerating invasion rate in a highly invaded estuary. Science, 279(5350), 555–558. 9438847 10.1126/science.279.5350.555

[ece33430-bib-0027] Copp, G. H. , Bianco, P. G. , Bogutskaya, N. G. , Eros, T. , Falka, I. , Ferreira, M. T. , … Wiesner, C. (2005). To be, or not to be, a non‐native freshwater fish? Journal of Applied Ichthyology, 21(4), 242–262.

[ece33430-bib-0028] Cranfield, H. J. , Gordon, D. P. , Willian, R. C. , Marshall, B. A. , Battershill, C. N. , Francis, M. P. , … Read, G. B. (1998). Adventive marine species in New Zealand. Technical report, NIWA Technical Report 34, 48 pp.

[ece33430-bib-0029] Creese, R. , Hooker, S. , De Luca, S. , & Wharton, Y. (1997). Ecology and environmental impact of *Musculista senhousia* (Mollusca: Bivalvia: Mytilidae) in Tamaki estuary, Auckland, New Zealand. New Zealand Journal of Marine and Freshwater Research, 31(2), 225–236.

[ece33430-bib-0030] Cremades, J. , Freire, O. , & Peteiro, C. (2006). Biologia, distribucion e integracion del alga aloctona *Undaria pinnatifida* (laminariales, phaeophyta) en las comunidades bentonicas de las costas de galicia (nw de la peninsula iberica). Anales del Jardin Botanico de Madrid, 63(2), 169–187.

[ece33430-bib-0031] Critchley, A. T. , Farnham, W. F. , & Morrell, S. L. (1986). An account of the attempted control of an introduced marine alga, *Sargassum muticum,* in Southern England. Biological Conservation, 35(4), 313–332.

[ece33430-bib-0032] Crooks, J. A. (2002). Characterizing ecosystem‐level consequences of biological invasions: the role of ecosystem engineers. Oikos, 97(2), 153–166.

[ece33430-bib-0033] Crooks, J. A. (2005). Lag times and exotic species: The ecology and management of biological invasions in slow‐motion. Ecoscience, 12(3), 316–329.

[ece33430-bib-0034] Crooks, J. A. , & Khim, H. S. (1999). Architectural vs. biological effects of a habitat‐altering, exotic mussel, *Musculista senhousia* . Journal of Experimental Marine Biology and Ecology, 240(1), 53–75.

[ece33430-bib-0035] Curiel, D. , Guidetti, P. , Bellemo, G. , Scattolin, M. , & Marzocchi, M. (2001). The introduced alga *Undaria pinnatifida* (Laminariales, Alariaceae) in the lagoon of Venice. Hydrobiologia, 477(1–3), 209–219.

[ece33430-bib-0036] DAISIE (2009). Handbook of Alien species in Europe, volume 3 of invading nature – Springer series in invasion ecology. Netherlands: Springer, 399 pp.

[ece33430-bib-0037] Davis, M. A. , Chew, M. K. , Hobbs, R. J. , Lugo, A. E. , Ewel, J. J. , Vermeij, G. J. , … Briggs, J. C. (2011). Don't judge species on their origins. Nature, 474(7350), 153–154. 21654782 10.1038/474153a

[ece33430-bib-0038] Dayton, P. K. (1985). Ecology of kelp communities. Annual Review of Ecology and Systematics, 16, 215–245.

[ece33430-bib-0039] De Leij, R. , Epstein, G. , Brown, M. , & Smale, D. A. (2017). The inuence of native macroalgal canopies on the distribution and abundance of the non‐native kelp *Undaria pinnatifida* in natural reef habitats. Marine Biology, 164, 156–171.

[ece33430-bib-0040] Dean, P. R. , & Hurd, C. L. (2007). Seasonal growth, erosion rates, and nitrogen and photo‐synthetic ecophysiology of *Undaria pinnatifida* (Heterokontophyta) in Southern New Zealand. Journal of Phycology, 43(6), 1138–1148.

[ece33430-bib-0041] Dellatorre, F. G. , Amoroso, R. , Saravia, J. , & Orensanz, J. M. (2014). Rapid expansion and potential range of the invasive kelp *Undaria pinnatifida* in the Southwest Atlantic. Aquatic Invasions, 9(4), 467–478.

[ece33430-bib-0042] Didham, R. K. , Tylianakis, J. M. , Hutchison, M. A. , Ewers, R. M. , & Gemmell, N. J. (2005). Are invasive species the drivers of ecological change? TRENDS in Ecology and Evolution, 20(9), 470–474. 16701420 10.1016/j.tree.2005.07.006

[ece33430-bib-0043] Dijkstra, J. A. , Harris, L. G. , Mello, K. , Litterer, A. , Wells, C. , & Ware, C. (2017). Invasive seaweeds transform habitat structure and increase biodiversity of associated species. Journal of Ecology, https://doi.org/10.1111/1365-2745.12775

[ece33430-bib-0044] Duyck, P. F. , David, P. , & Quilici, S. (2007). Can more K‐selected species be better invaders? A case study of fruit flies in La Runion. Diversity and Distributions, 13(5), 535–543.

[ece33430-bib-0045] Edgar, G. J. , Barrett, N. S. , Morton, A. J. , & Samson, C. R. (2004). Effects of algal canopy clearance on plant, fish and macroinvertebrate communities on eastern Tasmanian reefs. Journal of Experimental Marine Biology and Ecology, 312(1), 67–87.

[ece33430-bib-0046] Eno, N. C. , Clark, R. A. , & Sanderson, W. G. (1997). Non‐native marine species in British waters: a review and directory (p. 136). Peterborough, UK: Joint Nature Conservation Committee.

[ece33430-bib-0047] ES (2016). Proposal for a fiordland marine regional pathway management plan under the biosecurity act 1993. Report, Environment Southland, 71 pp.

[ece33430-bib-0048] Escapa, M. , Isacch, J. P. , Daleo, P. , Alberti, J. , Iribarne, O. , Borges, M. , … Lasta, M. (2004). The distribution and ecological effects of the introduced pacific oyster *Crassostrea gigas* (Thunberg, 1793) in northern Patagonia. Journal of Shellfish Research, 23(3), 765–772.

[ece33430-bib-0049] Eustice, G. (2014). Prohibition of keeping or release of live fish (specified species) (England) order 2014. Statutory Instrument No. 143, 4 pp.

[ece33430-bib-0050] Ewel, J. J. , O'Dowd, D. J. , Bergelson, J. , Daehler, C. C. , D'Antonio, C. M. , Gmez, L. D. , … Vitousek, P. M. (1999). Deliberate introductions of species: Research needs. Benefits can be reaped, but risks are high. BioScience, 49(8), 619–630.

[ece33430-bib-0051] Farrell, P. , & Fletcher, R. L. (2006). An investigation of dispersal of the introduced brown alga *Undaria pinnatifida* (Harvey) Suringar and its competition with some species on the man made structures of Torquay marina (Devon, UK). Journal of Experimental Marine Biology and Ecology, 334(2), 236–243.

[ece33430-bib-0052] Feldman, K. L. , Armstrong, D. A. , Dumbauld, B. R. , DeWitt, T. H. , & Doty, D. C. (2000). Oysters, crabs and burrowing shrimp: Review of an environmental conflict over aquatic resources and pesticide use in Washington State's (USA) coastal estuaries. Estuaries, 23(2), 141–176.

[ece33430-bib-0053] Fletcher, R. L. , & Farrell, P. (1999). Introduced brown algae in the North East Atlantic, with particular respect to *Undaria pinnatifida* (Harvey) Suringar. Helgolander Meeresuntersuchungen, 52(3–4), 259–275.

[ece33430-bib-0054] Floc'h, J.‐Y. , Pajot, R. , & Mouret, V. (1996). *Undaria pinnatifida* (laminariales, phaeophyta) 12 years after its introduction into the Atlantic Ocean. Hydrobiologia, 326(327), 217–222.

[ece33430-bib-0055] Floc'h, J. Y. , Pajot, R. , & Wallentinus, I. (1991). The Japanese brown alga *Undaria pinnatifida* on the coast of France and its possible establishment in European waters. Journal du Conseil – Conseil International pour l'Exploration de la Mer, 47(3), 379–390.

[ece33430-bib-0056] Forrest, B. M. , Brown, S. N. , Taylor, M. D. , Hurd, C. L. , & Hay, C. H. (2000). The role of natural dispersal mechanisms in the spread of *Undaria pinnatifida* (Laminariales, Phaeophyceae). Phycologia, 39(6), 547–553.

[ece33430-bib-0057] Forrest, B. M. , Gardner, J. P. A. , & Taylor, M. D. (2009). Internal borders for managing invasive marine species. Journal of Applied Ecology, 46(1), 46–54.

[ece33430-bib-0058] Forrest, B. M. , & Taylor, M. D. (2002). Assessing invasion impact: survey design considerations and implications for management of an invasive marine plant. Biological Invasions, 4, 375–386.

[ece33430-bib-0059] Gao, X. , Endo, H. , Taniguchi, K. , & Agatsuma, Y. (2013). Combined effects of seawater temperature and nutrient condition on growth and survival of juvenile sporophytes of the kelp *Undaria pinnatifida* (Laminariales; Phaeophyta) cultivated in northern Honshu, Japan. Journal of Applied Phycology, 25(1), 269–275.

[ece33430-bib-0060] Gao, X. , Endo, H. , Taniguchi, K. , & Agatsuma, Y. (2014). Effects of experimental thinning on the growth and maturation of the brown alga *Undaria pinnatifida* (Laminariales; Phaeophyta) cultivated in Matsushima bay, northern Japan. Journal of Applied Phycology, 26(1), 529–535.

[ece33430-bib-0061] Gerard, V. A. (1982). In situ water motion and nutrient uptake by the giant kelp *Macrocystis pyrifera* . Marine Biology, 69(1), 51–54.

[ece33430-bib-0062] Gittenberger, A. (2007). Recent population expansions of non‐native ascidians in the Netherlands. Journal of Experimental Marine Biology and Ecology, 342(1), 122–126.

[ece33430-bib-0063] Gittenberger, A. , & Stegenga, H. (2013). Risico analyse van uitheemse soorten in de export gebieden voor zuid ‐ noord transporten van de oosterschelde naar de waddenzee. Report, Producentenorganisatie van de Nederlandse Mosselcultuur, 8 pp.

[ece33430-bib-0064] Gozlan, R. E. (2008). Introduction of non‐native freshwater fish: Is it all bad? Fish and Fisheries, 9(1), 106–115.

[ece33430-bib-0065] Green, S. J. , Akins, J. L. , Maljkovi, A. , & Ct, I. M. (2012). Invasive lionfish drive Atlantic coral reef fish declines. PLoS ONE, 7(3), e32596. 22412895 10.1371/journal.pone.0032596 PMC3296711

[ece33430-bib-0066] Grosholz, E. (2002). Ecological and evolutionary consequences of coastal invasions. Trends in Ecology & Evolution, 17(1), 22–27.

[ece33430-bib-0067] Gurevitch, J. , & Padilla, D. K. (2004). Are invasive species a major cause of extinctions? Trends in Ecology & Evolution, 19(9), 470–474. 16701309 10.1016/j.tree.2004.07.005

[ece33430-bib-0068] Guy, C. , & Roberts, D. (2010). Can the spread of non‐native oysters (*Crassostrea gigas*) at the early stages of population expansion be managed? Marine Pollution Bulletin, 60(7), 1059–1064. 20189606 10.1016/j.marpolbul.2010.01.020

[ece33430-bib-0069] Harries, D. B. , Harrow, S. , Wilson, J. R. , Mair, J. M. , & Donnan, D. W. (2007). The establishment of the invasive alga *Sargassum muticum* on the west coast of Scotland: a preliminary assessment of community effects. Journal of the Marine Biological Association of the UK, 87, 1057–1067.

[ece33430-bib-0070] Hay, C. H. (1990). The dispersal of sporophytes of *Undaria pinnatifida* by coastal shipping in New Zealand, and implications for further dispersal of *Undaria* in France. British Phycological Journal, 25(4), 301–313.

[ece33430-bib-0071] Hay, C. H. , & Luckens, P. A. (1987). The Asian kelp *Undaria pinnatifida* (Phaeophyta: Laminariales) found in a New Zealand Harbour. New Zealand Journal of Botany, 25(2), 329–332.

[ece33430-bib-0072] Hay, C. H. , & Villouta, E. (1993). Seasonality of the adventive Asian kelp *Undaria pinnatifida* in New Zealand. Botanica Marina, 36(5), 461–476.

[ece33430-bib-0073] Heiser, S. , Hall‐Spencer, J. M. , & Hiscock, K. (2014). Assessing the extent of establishment of *Undaria pinnatifida* in Plymouth sound special area of conservation, UK. Marine Biodiversity Records, 7, e93.

[ece33430-bib-0074] Henkel, S. K. , & Hofmann, G. E. (2008). Thermal ecophysiology of gametophytes cultured from invasive *Undaria pinnatifida* (Harvey) Suringar in coastal California harbors. Journal of Experimental Marine Biology and Ecology, 367(2), 164–173.

[ece33430-bib-0075] Hewitt, C. L. (2003). Marine biosecurity issues in the world oceans: Global activities and Australian directions. Ocean Yearbook Online, 17(1), 193–212.

[ece33430-bib-0076] Hewitt, C. L. , Campbell, M. L. , McEnnulty, F. , Moore, K. M. , Murfet, N. B. , Robertson, B. , & Schaffelke, B. (2005). Efficacy of physical removal of a marine pest: the introduced kelp *Undaria pinnatifida* in a Tasmanian marine reserve. Biological Invasions, 7(2), 251–263.

[ece33430-bib-0077] Hobbs, R. J. , & Humphries, S. E. (1995). An integrated approach to the ecology and management of plant invasions. Conservation Biology, 9(4), 761–770.

[ece33430-bib-0078] Hulme, P. E. (2006). Beyond control: Wider implications for the management of biological invasions. Journal of Applied Ecology, 43(5), 835–847.

[ece33430-bib-0079] Irigoyen, A. J. , Eyras, C. , & Parma, A. M. (2010). Alien algae *Undaria pinnatifida* causes habitat loss for rocky reef fishes in North Patagonia. Biological Invasions, 13(1), 17–24.

[ece33430-bib-0080] Irigoyen, A. J. , Trobbiani, G. , Sgarlatta, M. P. , & Raffo, M. P. (2011). Effects of the alien algae *Undaria pinnatifida* (Phaeophyceae, Laminariales) on the diversity and abundance of benthic macrofauna in Golfo Nuevo (Patagonia, Argentina): potential implications for local food webs. Biological Invasions, 13(7), 1521–1532.

[ece33430-bib-0081] James, K. , Kibele, J. , & Shears, N. T. (2015). Using satellite‐derived sea surface temperature to predict the potential global range and phenology of the invasive kelp *Undaria pinnatifida* . Biological Invasions, 17(12), 3393–3408.

[ece33430-bib-0082] James, K. , Middleton, I. , Middleton, C. , & Shears, N. (2014). Discovery of *Undaria pinnatifida* (Harvey) Suringar, 1873 in northern New Zealand indicates increased invasion threat in subtropical regions. BioInvasions Records, 3(1), 21–24.

[ece33430-bib-0083] James, K. , & Shears, N. T. (2016a). Population ecology of the invasive kelp *Undaria pinnatifida* towards the upper extreme of its temperature range. Marine Biology, 163, 34.

[ece33430-bib-0084] James, K. , & Shears, N. T. (2016b). Proliferation of the invasive kelp *Undaria pinnatifida* at aquaculture sites promotes spread to coastal reefs. Marine Biology, 163, 225.

[ece33430-bib-0085] Jimenez, R. S. , Hepburn, C. D. , Hyndes, G. A. , McLeod, R. J. , Taylor, R. B. , & Hurd, C. L. (2015). Do native subtidal grazers eat the invasive kelp *Undaria pinnatifida*? Marine Biology, 162, 2521–2526.

[ece33430-bib-0086] Jormalainen, V. , Gagnon, K. , Sjroos, J. , & Rothusler, E. (2016). The invasive mud crab enforces a major shift in a rocky littoral invertebrate community of the Baltic Sea. Biological Invasions, 18(5), 1409–1419.

[ece33430-bib-0087] Jousson, O. , Pawlowski, J. , Zaninetti, L. , Zechman, F. W. , Dini, F. , Di Giuseppe, G. , … Meinesz, A. (2000). Invasive alga reaches California: The alga has been identified that threatens to smother Californian coastal ecosystems. Nature, 408(6809), 157–158. 11089959 10.1038/35041623

[ece33430-bib-0088] Kaplanis, N. J. , Harris, J. L. , & Smith, J. E. (2016). Distribution patterns of the non‐native seaweeds *Sargassum horneri* (turner) C. Agardh and *Undaria pinnatifida* (Harvey) Suringar on the San Diego and Pacific coast of North America. Aquatic Invasions, 11(2), 111–124.

[ece33430-bib-0089] Kim, Y. D. , Ahn, J. K. , Nam, M. M. , Lee, C. , Yoo, H. I. , Yeon, S. Y. , … Choi, J. S. (2016). Characteristics of algal succession following rock scraping at Imwon area in the east coast of Korea. Journal of Ocean University of China, 15(6), 1087–1093.

[ece33430-bib-0090] Kim, Y. S. , & Nam, K. W. (1997). Temperature and light responses on the growth and maturation of gametophytes of *Undaria pinnatifida* (Harvey) Suringar in Korea. Journal of the Korean Fisheries Society, 30, 505–510.

[ece33430-bib-0091] Koh, C. H. , & Shin, H. C. (1990). Growth and size distribution of some large brown algae in Ohori, east coast of Korea. Hydrobiologia, 204–205(1), 225–231.

[ece33430-bib-0092] Kolar, C. S. , & Lodge, D. M. (2001). Progress in invasion biology: Predicting invaders. TRENDS in Ecology and Evolution, 16(4), 199–204. 11245943 10.1016/s0169-5347(01)02101-2

[ece33430-bib-0093] Leliaert, F. , Kerckhof, F. , & Coppejans, E. (2000). Eerste waarnemingen van *Undaria pinnatifida* (Harvey) Suringar (Laminariales, Phaeophyta) en de epifyt pterothamnion plumula (Ellis) Nageli (Ceramiales, Rhodophyta) in Noord Frankrijk en belgie. Het Zeepard, 59, 71–73.

[ece33430-bib-0094] Levine, J. M. , & D'Antonio, C. M. (1999). Elton revisited: A review of evidence linking diversity and invasibility. Oikos, 87(1), 15–26.

[ece33430-bib-0095] Lockwood, J. L. , Cassey, P. , & Blackburn, T. (2005). The role of propagule pressure in explaining species invasions. TRENDS in Ecology and Evolution, 20(5), 223–228. 16701373 10.1016/j.tree.2005.02.004

[ece33430-bib-0096] Lodge, D. M. (1993). Biological invasions: Lessons for ecology. Trends in Ecology & Evolution, 8(4), 133–137. 21236129 10.1016/0169-5347(93)90025-K

[ece33430-bib-0097] Lowe, S. , Browne, M. , Boudjekas, S. , & De Poorter, M. (2000). 100 of the World's Worst Invasive Alien Species. The Invasive Species Specialist Group (ISSG) a specialist group of the Species Survival Commission (SSC) of the World Conservation Union (IUCN), 11 pp.

[ece33430-bib-0098] Lugo, A. E. (2004). The outcome of alien tree invasions in Puerto Rico. Frontiers in Ecology and the Environment, 2(5), 265–273.

[ece33430-bib-0099] MacDougall, A. S. , & Turkington, R. (2005). Are invasive species drivers or passengers of change in degraded ecosystems. Ecology, 86(1), 42–55.

[ece33430-bib-0100] MAF (2010). The commercial use of Undaria pinnatifida an exotic Asian seaweed. Report Paper No: 2010/02, Ministry of Agriculture and Forestry, 11 pp.

[ece33430-bib-0101] Martin, J. P. , & Bastida, R. (2008). The invasive seaweed *Undaria pinnatifida* (Harvey) Suringar in Ria Deseado (southern Patagonia, Argentina): Sporophyte cycle and environmental factors determining its distribution. Revista De Biologia Marina Y Oceanografia, 43(2), 335–344.

[ece33430-bib-0102] Matsuyama, K. (1983). Photosynthesis of *Undaria pinnatifida* Suringar f. Distans Miyabe et Okamura (Phaeophyceae) from Oshoro bay. 1. Seasonal changes of photosynthetic and respiratory rates. Scientific Reports of Hokkaido Fisheries Experimental Station, 25, 187–194.

[ece33430-bib-0103] McKinney, M. L. , & Lockwood, J. L. (1999). Biotic homogenization: A few winners replacing many losers in the next mass extinction. TRENDS in Ecology and Evolution, 14(11), 450–453. 10511724 10.1016/s0169-5347(99)01679-1

[ece33430-bib-0104] Minchin, D. , Cook, E. J. , & Clark, P. F. (2013). Alien species in British brackish and marine waters. Aquatic Invasions, 8(1), 3–19.

[ece33430-bib-0105] Minchin, D. , & Nunn, J. (2014). The invasive brown alga *Undaria pinnatifida* (Harvey) Suringar, 1873 (Laminariales: Alariaceae), spreads northwards in Europe. BioInvasions Records, 3(2), 57–63.

[ece33430-bib-0106] Molnar, J. L. , Gamboa, R. L. , Revenga, C. , & Spalding, M. D. (2008). Assessing the global threat of invasive species to marine biodiversity. Frontiers in Ecology and the Environment, 6(9), 485–492.

[ece33430-bib-0107] Morelissen, B. , Dudley, B. D. , Geange, S. W. , & Phillips, N. E. (2013). Gametophyte reproduction and development of *Undaria pinnatifida* under varied nutrient and irradiance conditions. Journal of Experimental Marine Biology and Ecology, 448, 197–206.

[ece33430-bib-0108] Morelissen, B. , Dudley, B. D. , & Phillips, N. E. (2016). Recruitment of the invasive kelp *Undaria pinnatifida* does not always benefit from disturbance to native algal communities in low‐intertidal habitats. Marine Biology, 163, 241.

[ece33430-bib-0109] Morita, T. , Kurashima, A. , & Maegawa, M. (2003a). Temperature requirements for the growth and maturation of the gametophytes of *Undaria pinnatifida* and *U. undarioides* (Laminariales, Phaeophyceae). Phycological Research, 51(3), 154–160.

[ece33430-bib-0110] Morita, T. , Kurashima, A. , & Maegawa, M. (2003b). Temperature requirements for the growth of young sporophytes of *Undaria pinnatifida* and *Undaria* undarioides (Laminariales, Phaeophyceae). Phycological Research, 51(4), 266–270.

[ece33430-bib-0111] Morrison, K. D. , Reekie, E. G. , & Jensen, K. I. N. (1998). Biocontrol of common St. Johnswort (hypericum perforatum) with *Chrysolina hyperici* and a host‐specific Colletotrichum gloeosporioides. Weed Technology, 12(3), 426–435.

[ece33430-bib-0112] Nanba, N. , Fujiwara, T. , Kuwano, K. , Ishikawa, Y. , Ogawa, H. , & Kado, R. (2011). Effect of water flow velocity on growth and morphology of cultured *Undaria pinnatifida* sporophytes (Laminariales, Phaeophyceae) in Okirai bay on the Sanriku coast, Northeast Japan. Journal of Applied Phycology, 23(6), 1023–1030.

[ece33430-bib-0113] Newsome and Noble (1986) Ecological and physiological characters of invading species. In: Ecology of Biological Invasions. Eds Groves R H and Burdon J J Cambridge University Press, Cambridge, UK p1‐20

[ece33430-bib-0114] NSPMMPI (2015). The national system for the prevention and management of marine pest in cursions – Australian Emergency marine pest plan (empplan) rapid response manual Undaria pinnatifida. Report, Australian Government – The National System for the Prevention and Management of Marine Pest Incursions, 52 pp.

[ece33430-bib-0115] NSW (1994). Schedule 6C noxious fish and noxious marine vegetation. New South Wales Fisheries Management Act 1994 No. 38.

[ece33430-bib-0116] Nyberg, C. D. , & Wallentinus, I. (2005). Can species traits be used to predict marine macroalgal introductions? Biological Invasions, 7, 265–279.

[ece33430-bib-0117] Occhipinti‐Ambrogi, A. (2007). Global change and marine communities: Alien species and climate change. Marine Pollution Bulletin, 55(7–9), 342–352. 17239404 10.1016/j.marpolbul.2006.11.014

[ece33430-bib-0118] Olabarria, C. , Rodil, I. F. , Incera, M. , & Troncoso, J. S. (2009). Limited impact of *Sargassum muticum* on native algal assemblages from rocky intertidal shores. Marine Environmental Research, 67(3), 153–158. 19168211 10.1016/j.marenvres.2008.12.007

[ece33430-bib-0119] Paalvast, P. , van Wesenbeeck, B. K. , van der Velde, G. , & de Vries, M. B. (2012). Pole and pontoon hulas: An effective way of ecological engineering to increase productivity and biodiversity in the hard‐substrate environment of the port of Rotterdam. Ecological Engineering, 44, 199–209.

[ece33430-bib-0120] Padilla, D. K. (2010). Context‐dependent impacts of a non‐native ecosystem engineer, the pacific oyster *Crassostrea gigas* . Integrative and Comparative Biology, 50(2), 213–225. 21558200 10.1093/icb/icq080

[ece33430-bib-0121] Pang, S. , & Luning, K. (2004). Photoperiodic long‐day control of Sporophyll and hair formation in the brown alga *Undaria pinnatifida* . Journal of Applied Phycology, 16(2), 83–92.

[ece33430-bib-0122] Pang, S. , & Shan, T. (2008). Zoospores of *Undaria pinnatifida*: their efficiency to attach under different water velocities and conjugation behavior during attachment. Acta Oceanologica Sinica, 27(6), 94–101.

[ece33430-bib-0123] Pang, S. , & Wu, C. (1996). Study on gametophyte vegetative growth of *Undaria pinnatifida* and its applications. Chinese Journal of Oceanology and Limnology, 14(3), 205–210.

[ece33430-bib-0124] Parker, I. M. , Simberloff, D. , Lonsdale, W. M. , Goodell, K. , Wonham, M. , Kareiva, P. M. , … Goldwasser, L. (1999). Impact: Toward a framework for understanding the ecological effects of invaders. Biological Invasions, 1(1), 3–19.

[ece33430-bib-0125] Perez, R. , Lee, J. Y. , & Juge, C. (1981). Observations sur la biologie de l'algue japonaise *Undaria pinnatifida* (Harvey) suringar introduite accidentellement dans l'etang de thau. Science et Peche, 325, 1–12.

[ece33430-bib-0126] Perez‐Cirera, J. , Salinas, J. , Cremades, J. , Barbara, I. , Granja, A. , Veiga, A. , & Fuertes, C. (1997). Cultivo de *Undaria pinnatifida* (laminariales, phaeophyta) en galicia. Nova Acta Cientifica Compostelana (Bioloxia), 7, 3–28.

[ece33430-bib-0127] Perrings, C. , Burgiel, S. , Lonsdale, M. , Mooney, H. , & Williamson, M. (2010). International cooperation in the solution to trade‐related invasive species risks. Annals of the New York Academy of Sciences, 1195, 198–212. 20536824 10.1111/j.1749-6632.2010.05453.x

[ece33430-bib-0128] Peteiro, C. , & Freire, O. (2011). Effect of water motion on the cultivation of the commercial seaweed *Undaria pinnatifida* in a coastal bay of Galicia, northwest Spain. Aquaculture, 314(1–4), 269–276.

[ece33430-bib-0129] Peteiro, C. , & Sanchez, N. (2012). Comparing salinity tolerance in early stages of the sporophytes of a non‐indigenous kelp (*Undaria pinnatifida*) and a native kelp (Saccharina Latissima). Russian Journal of Marine Biology, 38(2), 197–200.

[ece33430-bib-0130] Peteiro, C. , Sanchez, N. , & Martinez, B. (2016). Mariculture of the Asian kelp *Undaria pinnatifida* and the native kelp Saccharina Latissima along the Atlantic coast of Southern Europe: An overview. Algal Research, 15, 9–23.

[ece33430-bib-0131] Pimentel, D. , Zuniga, R. , & Morrison, D. (2005). Update on the environmental and economic costs associated with alien‐invasive species in the United States. Ecological Economics 52 (3SPEC. ISS.), 273–288.

[ece33430-bib-0132] Primo, C. , Hewitt, C. L. , & Campbell, M. L. (2010). Reproductive phenology of the introduced kelp *Undaria pinnatifida* (Phaeophyceae, Laminariales) in Port Phillip bay (Victoria, Australia). Biological Invasions, 12(9), 3081–3092.

[ece33430-bib-0133] Raffo, P. M. , Eyras, C. M. , & Iribarne, O. O. (2009). The invasion of *Undaria pinnatifida* to a *Macrocystis pyrifera* kelp in Patagonia (Argentina, South‐West Atlantic). Journal of the Marine Biological Association of the United Kingdom, 89(8), 1571–1580.

[ece33430-bib-0134] Richardson, D. M. , Pysek, P. , & Carlton, J. T. (2011). A compendium of essential concepts and terminology in invasion ecology In Richardson D. M. (Ed.), Fifty years of invasion ecology: the legacy of Charles Elton (pp. 409–420). Sussex, UK: Blackwell Publishing Ltd.

[ece33430-bib-0135] Rodriguez, L. F. (2006). Can invasive species facilitate native species? Evidence of how, when, and why these impacts occur. Biological Invasions, 8(4), 927–939.

[ece33430-bib-0136] Ross, D. J. , Johnson, C. R. , & Hewitt, C. L. (2003). Assessing the ecological impacts of an introduced seastar: The importance of multiple methods. Biological Invasions, 5(1–2), 3–21.

[ece33430-bib-0137] Ruiz, G. M. , Carlton, J. T. , Grosholz, E. D. , & Hines, A. H. (1997). Global invasions of marine and estuarine habitats by non‐indigenous species: mechanisms, extent, and consequences. American Zoologist, 37(6), 621–632.

[ece33430-bib-0138] Russell, L. K. , Hepburn, C. D. , Hurd, C. L. , & Stuart, M. D. (2008). The expanding range of *Undaria pinnatifida* in southern New Zealand: distribution, dispersal mechanisms and the invasion of wave‐exposed environments. Biological Invasions, 10(1), 103–115.

[ece33430-bib-0139] Saito, Y. (1975). Advance of phycology in Japan, Book section Undaria (pp. 304–320). The Hague: VEB Gustav Fischer Verlag.

[ece33430-bib-0140] Sala, O. E. , Chapin Iii, F. S. , Armesto, J. J. , Berlow, E. , Bloomfield, J. , Dirzo, R. , … Wall, D. H. (2000). Global biodiversity scenarios for the year. Science, 287(5459), 1770–1774. 10710299 10.1126/science.287.5459.1770

[ece33430-bib-0141] Sanchez, I. , & Fernandez, C. (2005). Impact of the invasive seaweed *Sargassum muticum* (Phaeophyta) on an intertidal macroalgal assemblage. Journal of Phycology, 41(5), 923–930.

[ece33430-bib-0142] Schaffelke, B. , Campbell, M. L. , & Hewitt, C. L. (2005). Reproductive phenology of the introduced kelp *Undaria pinnatifida* (Phaeophyceae, Laminariales) in Tasmania, Australia. Phycologia, 44(1), 84–94.

[ece33430-bib-0143] Schaffelke, B. , & Hewitt, C. L. (2007). Impacts of introduced seaweeds. Botanica Marina, 50(5–6), 397–417.

[ece33430-bib-0144] Schiel, D. R. , & Thompson, G. A. (2012). Demography and population biology of the invasive kelp *Undaria pinnatifida* on shallow reefs in southern New Zealand. Journal of Experimental Marine Biology and Ecology, 434, 25–33.

[ece33430-bib-0145] Schlaepfer, M. A. , Sax, D. F. , & Olden, J. D. (2011). The potential conservation value of non‐native species. Conservation Biology, 25(3), 428–437. 21342267 10.1111/j.1523-1739.2010.01646.x

[ece33430-bib-0146] Schlaepfer, M. A. , Sax, D. F. , & Olden, J. D. (2012). Toward a more balanced view of non‐native species. Conservation Biology, 26(6), 1156–1158. 23082954 10.1111/j.1523-1739.2012.01948.x

[ece33430-bib-0147] Shpigel, M. , & Blaylock, R. A. (1991). The pacific oyster, *Crassostrea gigas*, as a biological filter for a marine fish aquaculture pond. Aquaculture, 92(C), 187–197.

[ece33430-bib-0148] Silva, P. C. , Woodfield, R. A. , Cohen, A. N. , Harris, L. H. , & Goddard, J. H. R. (2002). First report of the Asian kelp *Undaria pinnatifida* in the northeastern Pacific Ocean. Biological Invasions, 4(3), 333–338.

[ece33430-bib-0149] Simberloff, D. (2011). How common are invasion‐induced ecosystem impacts? Biological Invasions, 13(5), 1255–1268.

[ece33430-bib-0150] Sinner, J. , Forrest, B. M. , & Taylor, M. D. (2000). A strategy for managing the Asian kelp Undaria: Final Report, Volume No. 578 of Cawthorn Report. Cawthorn Institute, Nelson: Ministry of Fisheries, New Zealand, pp 130.

[ece33430-bib-0151] Skriptsova, A. , Khomenko, V. , & Isakov, V. (2004). Seasonal changes in growth rate, morphology and alginate content in *Undaria pinnatifida* at the northern limit in the Sea of Japan (Russia). Journal of Applied Phycology, 16, 17–21.

[ece33430-bib-0152] Sliwa, C. , Johnson, C. R. , & Hewitt, C. L. (2006). Mesoscale dispersal of the introduced kelp *Undaria pinnatifida* attached to unstable substrata. Botanica Marina, 49(5–6), 396–405.

[ece33430-bib-0153] South, P. M. , Lilley, S. A. , Tait, L. W. , Alestra, T. , Hickford, M. J. H. , Thomsen, M. S. , & Schiel, D. R. (2015). Transient effects of an invasive kelp on the community structure and primary productivity of an intertidal assemblage. Marine and Freshwater Research, 67(1), 103–112.

[ece33430-bib-0154] South, P. M. , & Thomsen, M. S. (2016). The ecological role of invading *Undaria pinnatifida*: an experimental test of the driver‐passenger models. Marine Biology, 163, 175.

[ece33430-bib-0155] Stachowicz, J. J. , Fried, H. , Osman, R. W. , & Whitlatch, R. B. (2002). Biodiversity, invasion resistance, and marine ecosystem function: Reconciling pattern and process. Ecology, 83(9), 2575–2590.

[ece33430-bib-0156] Staehr, P. A. , Pedersen, M. F. , Thomsen, M. S. , Wernberg, T. , & Krause‐Jensen, D. (2000). Invasion of *Sargassum muticum* in Limfjorden (Denmark) and its possible impact on the indigenous macroalgal community. Marine Ecology Progress Series, 207, 79–88.

[ece33430-bib-0157] Suarez‐Jimenez, R. , Hepburn, C. D. , Hyndes, G. A. , McLeod, R. J. , Taylor, R. B. , & Hurd, C. L. (2017). Importance of the invasive macroalga *Undaria pinnatifida* as trophic subsidy for a beach consumer. Marine Biology, 164(5), 113.

[ece33430-bib-0158] Suto, S. (1952). On shedding of zoospores in some algae of Laminariaceae‐II. Nippon Suisan Gakkaishi, 18(1), 1–5.

[ece33430-bib-0159] Thompson, G. A. , & Schiel, D. R. (2012). Resistance and facilitation by native algal communities in the invasion success of *Undaria pinnatifida* . Marine Ecology Progress Series, 468, 95–105.

[ece33430-bib-0160] Thomsen, M. S. , Olden, J. D. , Wernberg, T. , Griffin, J. N. , & Silliman, B. R. (2011). A broad framework to organize and compare ecological invasion impacts. Environmental Research, 111(7), 899–908. 21696719 10.1016/j.envres.2011.05.024

[ece33430-bib-0161] Thomsen, M. S. , Wernberg, T. , South, P. M. , & Schiel, D. R. (2016). Non‐native seaweeds drive changes in marine coastal communities around the world (pp. 147–185). Dordrecht: Springer Netherlands.

[ece33430-bib-0162] Thomsen, M. S. , Wernberg, T. , Tuya, F. , & Silliman, B. R. (2009). Evidence for impacts of nonindigenous macroalgae: A meta‐analysis of experimental field studies. Journal of Phycology, 45(4), 812–819. 27034210 10.1111/j.1529-8817.2009.00709.x

[ece33430-bib-0163] Thornber, C. S. , Kinlnan, B. P. , Graham, M. H. , & Stachowicz, J. J. (2004). Population ecology of the invasive kelp *Undaria pinnatifida* in California: environmental and biological controls on demography. Marine Ecology Progress Series, 268, 69–80.

[ece33430-bib-0164] Valentine, J. P. , & Johnson, C. R. (2003). Establishment of the introduced kelp *Undaria pinnatifida* in Tasmania depends on disturbance to native algal assemblages. Journal of Experimental Marine Biology and Ecology, 295(1), 63–90.

[ece33430-bib-0165] Valentine, J. P. , & Johnson, C. R. (2004). Establishment of the introduced kelp *Undaria pinnatifida* following dieback of the native macroalga phyllospora comosa in Tasmania. Australia. Marine and Freshwater Research, 55(3), 223–230.

[ece33430-bib-0166] Valentine, J. P. , & Johnson, C. R. (2005). Persistence of sea urchin (*Heliocidaris erythrogramma*) barrens on the east coast of Tasmania: Inhibition of macroalgal recovery in the absence of high densities of sea urchins. Botanica Marina, 48(2), 106–115.

[ece33430-bib-0167] Valentine, J. P. , Magierowski, R. H. , & Johnson, C. R. (2007). Mechanisms of invasion: Establishment, spread and persistence of introduced seaweed populations. Botanica Marina, 50(5–6), 351–360.

[ece33430-bib-0168] Veiga, P. , Torres, A. C. , Rubal, M. , Troncoso, J. , & Sousa‐Pinto, I. (2014). The invasive kelp *Undaria pinnatifida* (Laminariales, Ochrophyta) along the north coast of Portugal: Distribution model versus field observations. Marine Pollution Bulletin, 84(1–2), 363–365. 24910185 10.1016/j.marpolbul.2014.05.038

[ece33430-bib-0169] Verbrugge, L. , de Hoop, L. , Leuven, R. , Aukema, R. , Beringen, R. , Creemers, R. , … deHullu, E. (2015). Expertpanelbeoordeling van (potentile) risicos en managementopties van invasieve exoten in nederland. Report Inhoudelijke input voor het Nederlandse standpunt over de plaatsing van soorten op EU‐verordening 1143/2014., Bureau Risicobeoordeling & Onderzoeksprogrammering, Nederlandse Voedsel‐ enWarenautoriteit (NVWA), Ministerie van Economische Zaken, 54 pp.

[ece33430-bib-0170] Viejo, R. M. (1997). The effects of colonization by *Sargassum muticum* on Tidepool Macroalgal assemblages. Journal of the Marine Biological Association of the United Kingdom, 77, 325–340.

[ece33430-bib-0171] Vitule, J. R. S. , Freire, C. A. , Vazquez, D. P. , Nuez, M. A. , & Simberloff, D. (2012). Revisiting the potential conservation value of non‐native species. Conservation Biology, 26(6), 1153–1155. 23083005 10.1111/j.1523-1739.2012.01950.x

[ece33430-bib-0172] VLIZ (2011). Japanese kelp ‐ Undaria pinnatifida. Non‐native species in the Belgian part of the north sea and adjacent estuaries, alien species consortium, Anders marine institute. Retrieved from http://www.marinespecies.org/introduced/wiki/Japanse_kelp.

[ece33430-bib-0173] Watanabe, Y. , Nishihara, G. N. , Tokunaga, S. , & Terada, R. (2014). The effect of irradiance and temperature responses and the phenology of a native alga, *Undaria pinnatifida* (Laminariales), at the southern limit of its natural distribution in Japan. Journal of Applied Phycology, 26, 2405–2415.

[ece33430-bib-0174] Williams, F. , Eschen, R. , Harris, A. , Djeddour, D. , Pratt, C. , Shaw, R. S. , … Murphy, S. T (2010). The economic cost of invasive non‐native species on Great Britain, Volume CABI Project No. VM10066 Final Report. Wallingford, UK, 199 pp.

[ece33430-bib-0175] Williams, S. L. , & Smith, J. E. (2007). A global review of the distribution, taxonomy, and impacts of introduced seaweeds. Annual Review of Ecology, Evolution, and Systematics, 38, 327–359.

[ece33430-bib-0176] Williamson, M. H. , & Fitter, A. (1996). The characters of successful invaders. Biological Conservation, 78(1–2), 163–170.

[ece33430-bib-0177] Wood, C. A. , Bishop, J. D. D. , & Yunnie, A. L. E. (2015). Comprehensive Reassessment of NNS in Welsh marinas, Volume Grant GU9430. Welsh Government Resilient Ecosystems Fund (REF), 40 pp.

[ece33430-bib-0178] Wotton, D. M. , O'Brien, C. , Stuart, M. D. , & Fergus, D. J. (2004). Eradication success down under: heat treatment of a sunken trawler to kill the invasive seaweed *Undaria pinnatifida* . Marine Pollution Bulletin, 49(9–10), 844–849. 15530528 10.1016/j.marpolbul.2004.05.001

[ece33430-bib-0179] Yamanaka, R. , & Akiyama, K. (1993). Cultivation and utilization of *Undaria pinnatifida* (Wakame) as food. Journal of Applied Phycology, 5(2), 249–253.

[ece33430-bib-0180] Zabin, C. , Ashton, G. , Brown, C. , & Ruiz, G. (2009). Northern range expansion of the Asian kelp *Undaria pinnatifida* (Harvey) Suringar (Laminariales, Phaeophyceae) in western North America. Aquatic Invasions, 4(3), 429–434.

[ece33430-bib-0181] Zenni, R. D. , Lamy, J. B. , Lamarque, L. J. , & Port, A. J. (2014). Adaptive evolution and phenotypic plasticity during naturalization and spread of invasive species: Implications for tree invasion biology. Biological Invasions, 16(3), 635–644.

